# An Overview of Machine Learning within Embedded and Mobile Devices–Optimizations and Applications

**DOI:** 10.3390/s21134412

**Published:** 2021-06-28

**Authors:** Taiwo Samuel Ajani, Agbotiname Lucky Imoize, Aderemi A. Atayero

**Affiliations:** 1 Department of Electrical and Electronics Engineering, Faculty of Engineering, University of Lagos, Akoka 100213, Lagos State, Nigeria; taiwo.ajani.94@gmail.com; 2 Department of Electrical Engineering and Information Technology, Institute of Digital Communication, Ruhr University, 44801 Bochum, Germany; 3 Department of Electrical and Information Engineering, Covenant University, Ota 112233, Ogun State, Nigeria; atayero@cu.edu.ng

**Keywords:** embedded computing systems, computer architecture, mobile computing, machine learning, TinyML, deep learning, mobile devices, optimization techniques

## Abstract

Embedded systems technology is undergoing a phase of transformation owing to the novel advancements in computer architecture and the breakthroughs in machine learning applications. The areas of applications of embedded machine learning (EML) include accurate computer vision schemes, reliable speech recognition, innovative healthcare, robotics, and more. However, there exists a critical drawback in the efficient implementation of ML algorithms targeting embedded applications. Machine learning algorithms are generally computationally and memory intensive, making them unsuitable for resource-constrained environments such as embedded and mobile devices. In order to efficiently implement these compute and memory-intensive algorithms within the embedded and mobile computing space, innovative optimization techniques are required at the algorithm and hardware levels. To this end, this survey aims at exploring current research trends within this circumference. First, we present a brief overview of compute intensive machine learning algorithms such as hidden Markov models (HMM), *k*-nearest neighbors (*k*-NNs), support vector machines (SVMs), Gaussian mixture models (GMMs), and deep neural networks (DNNs). Furthermore, we consider different optimization techniques currently adopted to squeeze these computational and memory-intensive algorithms within resource-limited embedded and mobile environments. Additionally, we discuss the implementation of these algorithms in microcontroller units, mobile devices, and hardware accelerators. Conclusively, we give a comprehensive overview of key application areas of EML technology, point out key research directions and highlight key take-away lessons for future research exploration in the embedded machine learning domain.

## 1. Introduction

Embedded computing systems are fast proliferating every aspect of human endeavor today, finding useful application in areas such as wearable systems for health monitoring, wireless systems for military surveillance, networked systems as found in the internet of things (IoT), smart appliances for home automation, antilock braking systems in automobiles, amongst others [1]. Recent research trends in computing technology have seen a merger of machine learning methods and embedded computing for diverse applications. For example, to target the hostility and dynamism of mobile ad hoc networks (MANETs), Haigh et al. [2] explored enhancing the self-configuration of a MANET using machine learning techniques. Besides, the recent breakthroughs of deep learning models in application areas such as computer vision [3,4,5,6,7], speech recognition [8,9], language translation, and processing [10,11], robotics, and healthcare [12] make this overlap a key research direction for the development of next-generation embedded devices. Thus, this has opened a research thrust between embedded devices and machine learning models termed “Embedded Machine Learning” where machine learning models are executed within resource-constrained environments [13]. This research surveys key issues within this convergence of embedded systems and machine learning.

Machine learning methods such as SVMs for feature classification [14], CNNs for intrusion detection [15], and other deep learning techniques, require high computational and memory resources for effective training and inferencing [16,17,18,19]. General-purpose CPUs, even with their architectural modification over the years, including pipelining, deep cache memory hierarchies, multicore enhancements, etc., cannot meet the high computational demand of deep learning models. However, graphic processing units (GPUs), due to their high floating-point performance and thread-level parallelism, are more suitable for training deep learning models [13]. Extensive research is actively being carried out to develop suitable hardware acceleration units using FPGAs [20,21,22,23,24,25,26], GPUs, ASICs, and TPUs to create heterogeneous and sometimes distributed systems to meet up the high computational demand of deep learning models. At both the algorithm and hardware levels, optimization techniques for classical machine learning and deep learning algorithms are being investigated such as pruning, quantization, reduced precision, hardware acceleration, etc. to enable the efficient execution of machine learning models in mobile devices and other embedded systems [27,28,29]. 

The convergence of machine learning methods and embedded systems in which computationally intensive machine learning models target the resource-constrained embedded environment has opened a plethora of opportunities for research in computing technology. Although EML is just in its cradle, quite some work has been done to: (1) optimize different machine learning models to fit into resource-limited environments, (2) develop efficient hardware architectures (acceleration units) using custom chipsets to accelerate the implementation of these algorithms, and (3) create novel and innovative specialized hardware architectures to meet the high-performance requirements of these models. Thus, there is a need to bring these perspectives together to provide the interested researcher with the fundamental concepts of EML and further provide the computer architect with insights and possibilities within this space.

**Figure 1:**
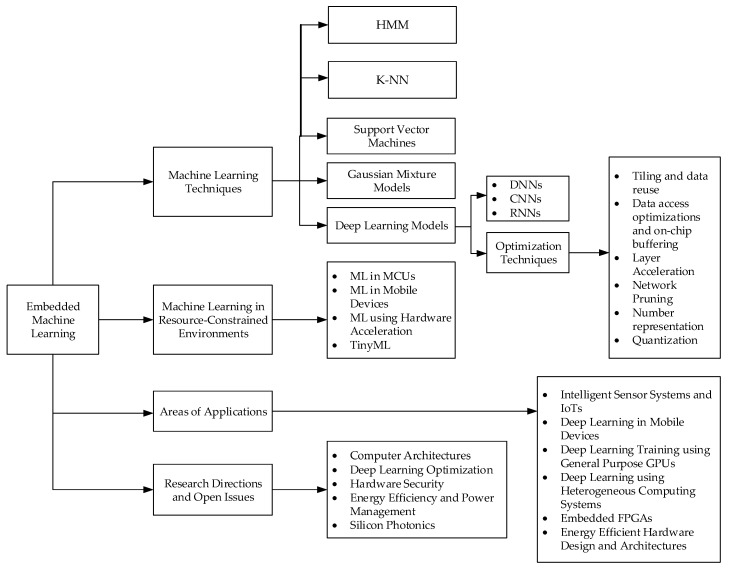
The layout of Embedded Machine Learning Computing Architectures and Machine Learning and Optimization Techniques.

Interestingly, several surveys have been carried out to achieve this. For example references [30,31] survey deep learning concepts, models, and optimizations. In these surveys, little consideration is given to the hardware architectural design, which is a key concern in developing efficient machine learning systems. Pooja [32] surveys recent trends in the hardware architectural design for machine learning applications, using the tensor processing unit as a case study. However, the research did not explore the different DNN architectures and just skimmed through some deep learning optimization techniques. Jiasi and Xukan [33], in their review, explored deep learning concepts, narrowing down on inference at end devices but do not compare different embedded chipset architectures to inform which architecture or optimization is appropriate for the different DNN models. They also present applications of deep learning in end devices. They, however, only explored a type of deep learning model (DNNs) and did not discuss other deep learning models (CNN, RNN), which have gained attention in recent times. Sergio et al. [24] have carried out a comprehensive survey on ML in embedded and mobile devices, presenting ML concepts and techniques for optimization and also investigated different application areas. They, however, also do not explore other models of DNNs or make appropriate trade-offs. To address these drawbacks, this survey presents key compute and memory intensive machine learning algorithms, which are the HMM, *k*-NN, SVM, GMM, and the different shades of DNNs (CNN and RNN), and present hardware-based and algorithm-based optimization techniques required to compress these algorithms within resource-constrained environments. To sum up, the authors decided to consider diverse application areas where machine learning has been utilized in proffering solutions to stringent problems in this big data era. A comprehensive layout of this survey is presented in Figure 1.

The key contributions of this survey are as follows:

- We present a survey of machine learning models commonly used in embedded systems applications.
- We describe an overview of compute-intensive machine learning models such as HMMs, *k*-NNs, SVMs, GMMs, and DNNs.
- We provide an overview of different optimization schemes adopted for these algorithms.
- We present an overview of the implementation of these algorithms within resource-limited environments such as MCUs, mobile devices, hardware accelerators, and TinyML.
- We survey the challenges faced in embedded machine learning and review different optimization techniques to enhance the execution of deep learning models within resource-constrained environments.
- We present diverse application areas of embedded machine learning, identify open issues and highlight key lessons learned for future research exploration.

The remainder of this paper is organized as follows. Section 2 presents embedded machine learning algorithms and specific optimization techniques, while Section 3 describes machine learning in resource-constrained environments (MCUs, mobile devices, acceleration units, and TinyML). Section 4 presents challenges and possible optimization opportunities in embedded machine learning. Section 5 provides diverse areas of applications of embedded machine learning technology, while Section 6 presents plausible research directions, open issues, and lessons learned. In Section 7, a concise conclusion is presented.

## 2. Embedded Machine Learning Techniques

Machine learning is a branch of artificial intelligence that describes techniques through which systems learn and make intelligent decisions from available data. Machine learning techniques can be classified under three major groups, which are supervised learning, unsupervised learning, and reinforcement learning as described in Table 1. In supervised learning, labeled data can be learned while in unsupervised learning, hidden patterns can be discovered from unlabeled data, and in reinforcement learning, a system may learn from its immediate environment through the trial and error method [34,35,36]. The process of learning is referred to as the *training phase* of the model and is often carried out using computer architectures with high computational resources such as multiple GPUs. After learning, the trained model is then used to make intelligent decisions on new data. This process is referred to as the *inference phase* of the implementation. The inference is often intended to be carried out within user devices with low computational resources such as IoT and mobile devices.

### 2.1. Scope of ML Techniques Overview

In recent times, machine learning techniques have been finding useful applications in various research areas and particularly in embedded computing systems. In this research, we surveyed recent works of literature concerning machine learning techniques implemented within resource-scarce environments such as mobile devices and other IoT devices between 2014 and 2020. We present the results of this survey in a tabular form given in Table 2. Our survey revealed that of all available machine learning techniques, SVMs, GMMs, DNNs, *k*-NNs, HMMs, decision trees, logistic regression, *k*-means, and naïve Bayes are common techniques adopted for embedded and mobile applications. Naïve Bayes and decision trees have low complexity in terms of computation and memory costs and thus do not require innovative optimizations as pointed out by Sayali and Channe [37]. Logistic regression algorithms are computationally cheaper than naïve Bayes and decision trees, meaning they have even lower complexity [38]. HMMs, *k*-NNs, SVMs, GMMs, and DNNs are however computationally and memory intensive and hence, require novel optimization techniques to be carried out to be efficiently squeezed within resource-limited environments. We have thus limited our focus to these compute intensive ML models and discuss state-of-the-art optimization techniques through which these algorithms may be efficiently implemented within resource-constrained environments.

### 2.2. Hidden Markov Models

Hidden Markov Model is an unsupervised machine learning technique based on augmenting the Markov chain [85]. The Markov chain is a technique that describes the probability of a sequence of events from a set of random variables. HMMs have been successfully adopted for speech recognition, activity recognition, and gesture tracking applications [86]. In [56], Patil and Thorat adopt HMM within an embedded device for detecting diseases in grapes. Charissa and Song-bae in [61] implemented HMM using a smartphone for recognizing human activities. HMMs are however compute and memory intensive and thus require some optimization techniques for effective execution in resource-limited environments [87].

#### 2.2.1. The HMM Algorithm

The HMM is an algorithm that extracts meaningful information from available data through observing a sequence of “hidden states” or “hidden classes” in the data and can subsequently make accurate predictions of future states based on the current state [85]. Five important components that make up the hidden Markov model are the number of states, number of distinct observations, the state transition model, observation model, and initial state distribution [85]. To determine the probability of observations, a forward algorithm is adopted, while to predict the sequence of hidden states in the available data, the Viterbi algorithm is used. The learning phase of the HMM is carried out using the Baum-Welch Algorithm or the forward-backward algorithm [86]. An overview of these problems and algorithms is given by Equations (1)–(3), as defined in Table 3.

(1) $$P(Z|X)=\overset{T}{\underset{i=1}{\prod }}P\left(z_{i}|x_{i}\right)$$

(2) $$v_{t}\left(j\right)=\underset{i=1}{\max}v_{t-1}\left(i\right)a_{ij}b_{j}\left(x_{t}\right)$$

(3) $$\arg\underset{\lambda }{\max}P\left(X;\lambda \right)=\arg\underset{\lambda }{\max}\underset{z}{\sum }P\left(X,Z;\lambda \right)$$

#### 2.2.2. Some HMM Optimization Schemes

Although HMMs are suitable for different applications, they require a large amount of computational and memory resources for efficient implementation. Embedded and mobile devices are however resource-scarce environments and thus require novel optimization schemes to be carried out for the efficient execution of HMMs. In [87], Toth and Nemeth presented an optimized HMM to target smartphone environments for speech synthesis. They optimize the HMM by selecting optimal parameters and implemented the model using fixed-point arithmetic instead of computation-intensive floating-point arithmetic. Fu et al. [88] proposed a series of optimization techniques including parameter reduction using decision tree-based clustering, model compression, feature size reduction, and fixed-point arithmetic implementation. Their optimized HMM target resource-scarce embedded platforms for speech synthesis. A list of optimizations is presented in Table 4.

### 2.3. k-Nearest Neighbours

*k*-NN is a non-parametric and lazy supervised machine technique often adopted for classification problems e.g., text categorization [89]. *k*-NN algorithms have been adopted in several embedded applications. For example, Hristo et al. [41] develop a mobile device fingerprinting system based on the *k*-NN algorithm. Additionally, Sudip et al. [76] develop a smartphone-based health monitoring system using the *k*-NN model. A smartphone-based data mining system is presented in [60], for fall detection using a *k*-NN algorithm. *k*-NN algorithms are also memory and compute intensive and require appropriate optimizations for resource-scarce environments.

#### 2.3.1. The k-NN Algorithm

The *k*-NN algorithm unlike other ML approaches is a lazy learner because it does not use specialized training data to generalize, rather it uses all available data to classify. It is also non-parametric because it does not make assumptions from available data [40]. The Algorithm is such that computes the distance between the input data and other data points and using a predefined “*k* value”, classification is done by estimating proximity. The important Equations (4) and (5) that describe the *k*-NN model are defined in Table 5.

(4) $$\hat{y}=\rho \overset{n}{\underset{i=1}{\sum }}\sigma \left(y_{i}\right)K\left(x,x_{i}\right)$$

(5) $$D\left(p,q\right)=\sqrt{{\left(p_{1}-q_{1}\right)}^{2}+{\left(p_{2}-q_{2}\right)}^{2}+\ldots +{\left(p_{n}-q_{n}\right)}^{2}}$$

#### 2.3.2. Some k-NN Optimization Schemes

*k*-NN models require the entire input data to be stored in memory for prediction to be done hence they are memory and compute intensive [70]. To improve the efficiency of the *k*-NN model, Li et al. [89] proposed an improved *k*-NN that uses different *k values* for different categories instead of a predefined fixed *k value*. Norouzi et al. [90] investigated the optimization of *k*-NN algorithms by mapping input features to binary codes which are very memory efficient. There have also been some hardware-oriented optimization schemes to accelerate *k*-NN models. Saikia et al. [91]. Mohsin and Perera [55] developed an acceleration unit to increase the execution speed of *k*-NN models in resource-scarce environments. Also, Gupta et al. [70] proposed a modified *k*-NN model termed ProtoNN which is a highly compressed *k*-NN model suitable for resource-limited IoT devices. Table 6 describes some *k*-NN optimization schemes.

### 2.4. Support Vector Machines 

Support vector machine is a supervised machine learning technique based on Vapnik’s statistical learning theory often adopted for object classification problems. SVMs have been successfully applied to regression, ranking, and clustering problems [92]. Also, SVMs have been useful in the prediction of the power and performance, auto-tuning, and runtime scheduling of high-performance applications [93]. In [2] for example, SVM is adopted in maintaining a near-optimal configuration of a MANET. Also, SVMs are used in the design and development of a low-cost and energy-efficient intelligent sensor [94]. SVMs are, however, computationally and memory intensive and thus require hardware acceleration units to be effectively executed in resource-limited situations. In [95], the FPGA hardware implementation of an SVM is surveyed with optimization techniques. 

#### 2.4.1. The SVM Algorithm

An SVM is a linear or non-linear classifier that can identify two distinct objects by separating them into two unique classes with high accuracy. The SVM is then trained, during which a hyperplane is developed to separate the data belonging to each unique class. These hyperplane samples are referred to as “support vectors,” which are then used to classify new data. The problem equation for training an SVM is given in Equation (6):

(6) $$\max W\left(\propto \right)=\overset{l}{\underset{i=1}{\sum }}\propto_{i}-\frac{1}{2}\overset{l}{\underset{i=1}{\sum }}\cdot \overset{l}{\underset{j=1}{\sum }}y_{i}y_{j}k\left(x_{i},x_{j}\right)\propto_{i}\propto_{j}$$

where $\propto_{i}\propto_{j}$ are Lagrange Multipliers, $k\left(x_{i},x_{j}\right)$ are the kernel functions, *x* and *y* are positions, *W* is the quadratic function.

The algorithm flow is given in the pseudo-code, as shown in Algorithm 1.
**Algorithm 1.** Pseudocode for training a support vector machine**Require:***X* and *y* loaded with training labeled data, α←0 or *α*
← partially trained SVM 1: *C*
← some value (10 for example) 2: **repeat** 3:  **for all**
$\left\{x_{i}y_{i}\right\},\;\left\{x_{i}y_{i}\right\}$
**do** 4:   Optimize $\propto_{i}\;and\;\propto_{j}$ 5:  **end for** 6: **until** no changes in α or other resource constraint criteria met **Ensure:** Retain only the support vectors ($\propto_{i}>0$)

The implementation of training, testing, and predicting phases of an SVM involves kernel functions that are the dot products and the Gaussian kernel functions. These computations make SVM computationally intensive. Additionally, having to train much data to inform accurate prediction makes SVM models memory intensive. Thus, efficient optimizations are required both at the hardware architecture and algorithm levels. To efficiently compute kernel functions, hardware acceleration units have been developed using FPGAs so that these computationally intensive operations can be moved to the hardware [92]. Also, at the algorithm level, a sequential minimal optimization method may be used to reduce the memory usage [96].

#### 2.4.2. Some SVM Optimizations Schemes

SVM techniques are computing and memory intensive and thus require appropriate optimization methods to be successfully executed in resource-constrained environments. Some works of literature have investigated the optimization of SVM models. The training of the SVM involves solving a quadratic programming (QP) problem, and thus to solve this problem optimally, optimization techniques involving chunking, decomposition or sequential minimal optimizations, etc. may be carried out to reduce the memory footprint required for training the model. For inference, bit precision techniques, Logarithm number representations, quantization, etc., are some optimization techniques that may be applied to fit SVM models within resource-constrained environments. In [94], Boni et al. develop a model selection technique targeted at reducing SVMs for resource-constrained environments. Table 7 presents a comprehensive list of SVM optimization schemes for both the training and classification phases. Interestingly, the Kernel selection also informs the computational requirement of SVM models. Some kernel types are the Laplacian kernel, the Gaussian kernel, the sigmoid kernel, the linear kernel, etc. Of these kernel types, the most suitable for resource-constrained environments is the Laplacian kernel because it can be implemented using shifters [97]. 

### 2.5. Gaussian Mixture Model

GMMs are density models capable of representing a large class of sample distributions. They are used in finding the traffic density patterns in a large set of data. This characteristic makes them suitable for analyzing large sensor data in IoT devices and biometric systems, particularly for speaker recognition systems [105]. In [50], GMM is adopted within an embedded board for analyzing the volume of sensor data at run time to monitor certain conditions of a system. Although GMMs are efficient, deep learning models pose a more effective method of analyzing raw sensor data.

#### 2.5.1. The GMM Algorithm

A GMM is a weighted sum of M component Gaussian densities as shown in Equations (7a) and (7b). The parameters of a GMM are retrieved during the training phase using an expectation-maximization (EM) algorithm or maximum a posteriori (MAP) estimation technique. The accuracy of a GMM hugely depends on the amount of computational power and memory bandwidth required to implement the model:

(7a) $$p\left(x|\lambda \right)=\sum_{i=1}^{M}w_{i\;}g\left(x|\mu_{i},\;\Sigma_{i}\right),$$

(7b) $$g\left(x{|\mu }_{i},\;\Sigma_{i}\right)=\frac{1}{{\left(2\pi \right)}^{D/2}{\left|\Sigma_{i}\right|}^{1/2}}\exp\left\{-\frac{1}{2}{\left(x-\mu_{i}\right)}^{\prime }\Sigma^{-1}{}_{i}\;\left(x-\mu_{i}\right)\right\},$$

$$\lambda =\left\{w_{i},\;\mu_{i},\;\Sigma_{i}\right\}\;\;\;\;i=1,\ldots ,M.$$

where *x* is a *D*-dimensional continuous-valued data vector (features), *i* = 1,…, *M*, are the mixture weights, p(x|λ) is the probability density function, and $g\left(x|\mu_{i},\;\Sigma_{i}\right)$ are the component Gaussian densities, $\mu_{i}$ is the mean vector, $\Sigma_{i}$ is the covariance matrix.

#### 2.5.2. Some GMM Optimization Schemes

GMMs are used in representing and modeling large volumes of data as in sensor data systems and in background modeling tasks [105]. This characteristic makes GMMs highly computationally and memory intensive, and unsuitable for real-time oriented applications. Some literature has explored optimization techniques for reducing the computational requirement and memory footprint of GMMs. Pushkar and Bharadwaj [106], propose an enhanced GMM algorithm by minimizing the floating-point computations, modifying the switching schedule, and automatically selecting the number of modes to target resource-constrained embedded environments. Additionally, Shen et al. in [107] propose an optimized GMM model based on compressive sensing to reduce the dimensionality of the data while still retaining the relevant information. This technique is computationally and memory efficient. In another publication, Salvadori et al. [39] proposed a GMM optimization technique based on integers to target processors with no floating point unit (FPU). This work showed low computation and a highly reduced memory footprint. A list of these optimization schemes is presented in Table 8.

### 2.6. Deep Learning Models

Deep learning models are machine-learning techniques that model the human brain [30]. They use a hierarchical array of layers to learn from available data and make new predictions based on the information they extract from the set of raw data [13,30,31]. The primary layer types that make up a deep learning model are pooling layers, convolutional layers, classifier layers, and local response normalization layers. The high accuracy of deep learning algorithms in various areas of applications has made them very attractive for use in recent times. However, hardware architectural computational power is pushing hard to meet up the computational demand of these models to inform their optimal implementation. In this survey, we explore the three main classes of deep learning models, which are DNNs, CNNs, and RNNs. Table 9 describes popular DNN models with their parameters.

Furthermore, like other machine learning models, deep learning models go through three phases; train, test, and predict. The training phase of deep learning models is carried out using a feedforward technique that entails sequentially passing data through the entire network for a prediction to be made and back-propagating the error through the network. The technique for backpropagation is called stochastic gradient descent (SGD), which adjusts the weights or synapses of each layer in the network using a non-linear activation function (tanh, sigmoid, rectified linear unit (ReLU)) [108,109,110]. Lin and Juan [111] carry out research where they explore the possibility of developing an efficient hardware architecture to accelerate the activation function of the network. The training process is often carried out many times for the model to efficiently learn, and then using the trained model, the prediction is made on new data. The training is very computationally and memory intensive and is often carried out offline using very high-performance computing resources mostly found in large data centers, while the inference targets low cost and resource-constrained environments.

#### 2.6.1. Convolution Layers

The convolution layers are the input layers of most DNNs, and they are used to extract characteristic features from a given input data using a set of filters. These filters are vector products (kernels), and their coefficients form the synaptic layer weights [112]. Convolution layers thus perform the major number of multiply and accumulate operations in the entire network, which means they are computationally intensive and are the major drawback to the real-time performance of deep learning models. Hardware acceleration units can be developed to accelerate these layers to reduce the latency of implementing deep learning models. The output neuron of a convolution is described in Equation (8):

(8) $$out{\left(x,y\right)}^{f_{0}}=\overset{N_{i}f}{\underset{f_{i}=0}{\sum }}\overset{K_{x}}{\underset{k_{x}=0}{\sum }}\overset{K_{y}}{\underset{k_{y}=0}{\sum }}w_{f_{i},f_{0}}\left(k_{x}k_{y}\right)*in{\left(x+k_{x},y+k_{y}\right)}^{f_{i}}$$

where $out{\left(x,y\right)}^{f_{0}}$ represent the output neuron and $in{\left(x,y\right)}^{f_{0}}$ represents the input neuron, in *x* and *y* directions, $w_{f_{i},f_{0}}\left(k_{x}k_{y}\right)$ represent the synaptic weight and $\left(k_{x}k_{y}\right)$ represent the kernel position, $N_{i}f$ are the input features. In addition, *f_i_* and *f*_0_ represent the input and output feature maps, respectively.

#### 2.6.2. Pooling Layers

The pooling layers are used in subsampling the feature maps obtained for convolutions and computing the maximum/average of neighboring neurons in the same feature map. In summary, this layer helps reduce the input layer dimensionality, thereby reducing the total number of inputs into subsequent layers and we traverse down the neural network [112]. The pooling layer has no synaptic weight attached. Some research prunes away this layer from the entire network to reduce computation time. The equation to evaluate a pooling layer is given in Equation (9):

(9) $$out\left(f_{0},x,y\right)=\underset{0\leq \left(k_{x},k_{y}\right)<K}{\max}/average\;\left(in\left(f_{0},x+k_{x,}y+k_{y}\right)\right)$$

where $out\left(f_{0},x,y\right)$ is the output neurons at positions *x* and *y*, *K* is the number of feature maps in *x* and *y* directions, $\left(k_{x},k_{y}\right)$ are kernel positions in *x* and *y* directions respectively and $f_{0}$ represents the output feature maps.

#### 2.6.3. Normalization Layers

These layers inform competition between neurons at the same location but in different feature maps. They perform a process very similar to lateral inhibition in biological neurons. Their values may be evaluated from Equation (10). Some research works skip this layer in DNN implementation to accelerate the entire network.

(10) $$out{\left(x,y\right)}^{f}=\frac{in{\left(x,y\right)}^{f}}{{\left(c+\propto \sum_{g=\max\left(0,f-\frac{k}{2}\right)}^{\min\left(N_{f}-1,\;f+\frac{k}{2}\right)}{\left(\propto {\left(x,y\right)}^{g}\right)}^{2}\right)}^{\beta }}$$

where $out{\left(x,y\right)}^{f}$ are output neurons, $in{\left(x,y\right)}^{f}$ are input neurons, $N_{f}$ are the input features, *f* and *g* are input and output feature maps respectively, *k* is the number of adjacent feature maps, and *c*, *α*, and *β* are constants.

#### 2.6.4. Fully-Connected Layers 

These are layers where all the output features are fully connected to all the input features using synaptic weights. Each output neuron is a weighted sum of all the input neurons. Equation (11) describes the value of each output neuron. These layers are often used for classification and output. Interestingly, because this layer is fed with the processed output features of the previous layers, i.e., the Convolution, Pooling, and Normalization layers, the input features are always lower than those for other layers i.e., they perform a reduced amount of matrix multiplications. However, the full connection using synaptic weights makes them very memory hungry as they make up for the largest share of all synaptic weights:

(11) $$out\left(j\right)=t\overset{N_{i}}{\underset{i=0}{\sum }}w_{ij}*in\left(i\right)$$

where “*t*” is the non-linear activation function, $N_{i}$ are the input features, $w_{ij}$ are the synaptic weights, *i* and *j* are the input and output feature maps respectively.

#### 2.6.5. Fully-Connected Deep Neural Networks

DNNs are a class of neural networks that are used for speech recognition applications, extracting high-level human behaviors, etc. The DNN architecture is such that all the layers in the network are fully connected, and the output layer with the highest activation value gives the required prediction. This characteristic feature makes them suitable for learning from unstructured data. Owing to the full connectivity of all the layers in the network, DNNs are computationally intensive than CNNs but highly memory intensive. More explicitly, because they have to perform routine multiply-accumulate computations, their computational logic is not complex but they require large enough memory to store the synapses and activation functions. Therefore, optimizations for FC DNNs concern more memory-centric thrusts like model compression, sparsity, pruning, and quantization. In [113], DNN models are developed to be implemented in mobile devices by distributing the computation across different processors within the mobile device. A fully connected DNN is also referred to as a multilayer perceptron (MLP).

#### 2.6.6. Convolutional Neural Networks

**Figure 2:**
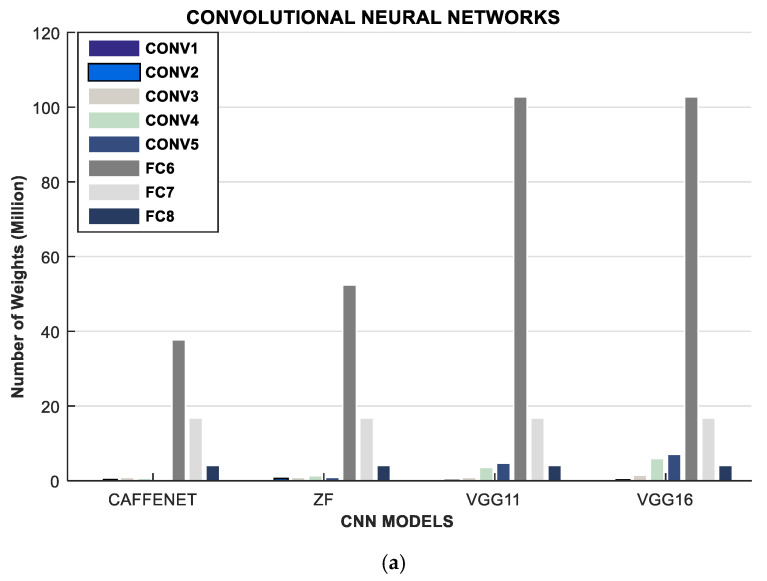

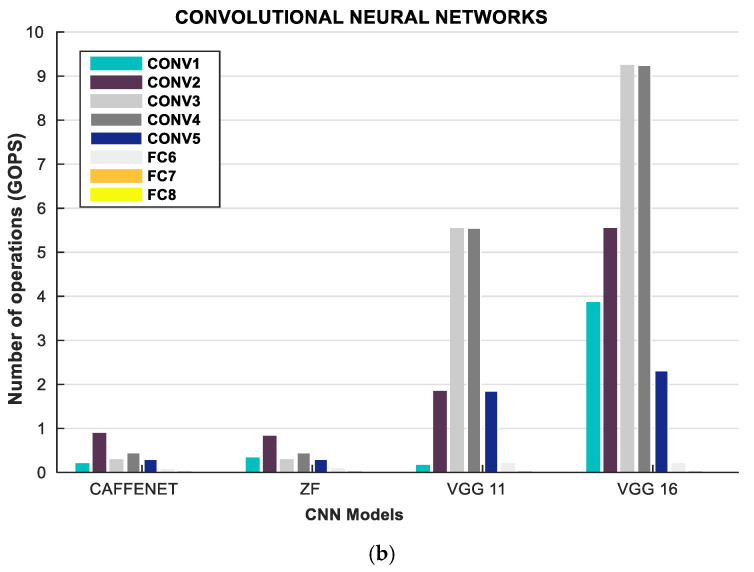
Description of a spectrum of certain CNN models to reveal their compute and memory demand: (**a**) Describes the Memory Demand of these models in terms of the number of weight parameters in (millions) (**b**) Computational Demand of these models in terms of their number of operations (GOPs).

CNNs are a class of neural networks that are suitable for computer vision applications, object recognition applications, etc. [114,115,116]. In ConvNets, key features in an image are extracted then converted into complex representations using the pooling layer, and subsequently, the fully-connected layers classify the image and identify the image appropriately. The CNN architecture is primarily made up of a large portion of convolution layers, followed by few fully connected layers. Some CNNs sometimes add pooling and normalization layers in-between the convolution and fully connected layers. The presence of convolution layers that perform kernel functions (vector-matrix multiplications) makes CNNs very computation-intensive and less memory-hungry due to the few fully connected layers. Therefore, optimizations for CNN models concern more computer-centric directions such as innovative hardware acceleration developments, processor technology, tiling, and data reuse, reduced precision, quantization, etc. Popular CNNs are ResNets, LeNets, AlexNets, GoogLeNets, VGGNets, etc. The computation and memory requirements of some CNN models are given in Figure 2 and summarized in Table 10.

#### 2.6.7. Recurrent Neural Networks

RNNs are a class of Neural Networks used for natural language translation applications, speech recognition, etc. RNNs, unlike CNNs, process input data sequentially and store the previous element. They thus retain and use past information which makes them suitable for text prediction applications and also for suggesting words in a sentence. The architecture of an RNN model is primarily made up of fully connected layers and normalization layers. This organization makes RNNs memory-centric in operation since they have to store weight synapses in available memory. A type of RNN referred to as Long Short Term Memory (LSTM) is gaining interest in recent times and process to be more effective than conventional RNNs [117,118,119,120]. Khan et al. [121] developed a data analytic framework with the real-world application using Spark Machine Learning and LSTM techniques. There are great hardware acceleration opportunities in implementing RNNs [122,123,124], and [23] explores the FPGA acceleration of an RNN. Table 11 describes some of the DNN models and their architecture.

## 3. Machine Learning in Resource-Constrained Environments

Machine learning techniques are currently targeting resource-scarce environments such as mobile devices, embedded devices, and other internet of things devices. In this section, we present an overview of different resource-limited environments like microcontroller units (MCUs) and mobile devices. We also discuss the option of hardware acceleration units used to speed up the execution of these algorithms in resource-scarce environments. 

### 3.1. Machine Learning Using Microcontrollers

Microcontrollers are at the front end of provisional hardware to implement diverse embedded systems and other IoT applications [126]. An MCU consists of a microprocessor, memory, I/O ports, and other peripherals all integrated into one chip. At the processor core of MCUs are general-purpose CPUs for adequate computation. Table 12 describes a list of popular MCUs using their compute and memory resources. From this list, we can observe the resource limitation of most MCUs in terms of available power and on-chip memory (flash + SRAM). This resource limitation is a critical drawback in the implementation of machine learning models. For example, some typical CNN model sizes are AlexNet (240 MB) [27], VGG 16 (528 MB) [127], VGG 19 (549 MB) [127], etc. Model size describes the number of bytes required to store all the parameters of the model. In this study, we survey techniques for compressing these models to fit into the available memory of MCUs for efficient computation to be appropriately done.

Table 12 presents a list of different microcontrollers categorized using their clock frequency, available flash memory, SRAM, and their current consumption. As can be observed in this table, microcontrollers have very limited hardware resources. This scarcity of resources makes them unsuitable for high-end machine learning applications, except the machine learning models are heavily optimized to fit within this space [24].

### 3.2. Machine Learning Using Hardware Accelerators

General-purpose CPU chipsets, although ubiquitous, do not possess enough computational ability to process compute-intensive ML algorithms. To address this drawback, hardware acceleration units may be developed using GPUs, FPGAs, and even ASICs. However, the most popular accelerators are FPGA-based accelerators owing to the programmability of FPGAs. Hardware acceleration is developed such that compute-intensive segments of the ML algorithm, e.g., kernel operations, are offloaded to the specialized accelerator, thereby relieving the CPU to process much simpler operations, improving the overall computation speed of the system. Some machine learning accelerators are Arm Ethos NPUs, Intel’s Movidus NCS, Nvidia’s Jetson nano, etc. Table 13 presents some of these accelerators. A critical drawback in adopting these accelerators is cost. Also, some works of literature have explored the development of FPGA-based accelerators for machine learning algorithms. Wang et al. [22] proposed a scalable deep learning accelerator to accelerate the kernel computations of deep learning. The authors introduce some optimization schemes such as tiling techniques, pipelining, FIFO buffers, and data reuse to further improved their proposed architecture.

### 3.3. Machine Learning in Mobile Devices 

Machine learning techniques are gradually permeating mobile devices for applications such as speech recognition, computer vision, etc. Mobile devices may be categorized under resource-constrained systems owing to their limited computational and memory resources. Hence, for machine learning algorithms to be successfully implemented within these devices, appropriate optimizations must be carried out. Lane et al. [137] developed a software accelerator for accelerating the execution of deep learning models within mobile devices. We present a survey of some mobile machine learning applications in the literature as tabulated in Table 14. 

### 3.4. TinyML

Machine learning inference at the edge particularly within very low power MCUs is gaining increased interest amongst the ML community. This interest pivots on creating a suitable platform where ML models may be efficiently executed within IoT devices. This has thus opened a growing research area in embedded machine learning termed TinyML. TinyML is a machine learning technique that integrates compressed and optimized machine learning to suit very low-power MCUs [141]. TinyML primarily differs from cloud machine learning (where compute intensive models are implemented using high-end computers in large datacenters like Facebook [142]), Mobile machine learning in terms of their very low power consumption (averagely 0.1 W) as shown in Table 15. TinyML creates a platform whereby machine learning models are pushed to user devices to inform good user experience for diverse applications and it has advantages such as energy efficiency, reduced costs, data security, low latency, etc., which are major concerns in contemporary cloud computing technology [141]. Colby et al. [143] presented a survey where neural network architectures (MicroNets) target commodity microcontroller units. The authors efficiently ported MicroNets to MCUs using the TensorFlow Lite Micro platform. There are different platforms developed to easily port ML algorithms to resource-constrained environments. Table 16 presents a list of available TinyML frameworks commonly adopted to push ML models into different compatible resource-limited devices.

## 4. Challenges and Optimization Opportunities in Embedded Machine Learning

Embedded computing systems are generally limited in terms of available computational power and memory requirements. Furthermore, they are required to consume very low power and to meet real-time constraints. Thus, for these computationally intensive machine learning models to be executed efficiently in the embedded systems space, appropriate optimizations are required both at the hardware architecture and algorithm levels [148,149]. In this section, we survey optimization methods to tackle bottlenecks in terms of power consumption, memory footprint, latency concerns, and throughput and accuracy loss.

### 4.1. Power Consumption 

**Figure 3:**
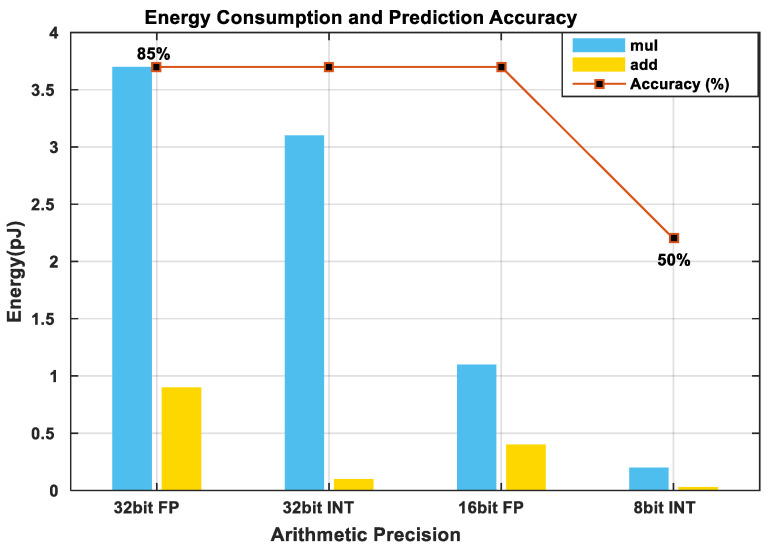
This graph describes the energy consumption and prediction accuracy of a DNN as a function of the Arithmetic Precision adopted for a single MAC unit in a 45 nm CMOS [124]. It may be deduced from the graph that lower number precisions consume less power than high precisions with no loss in prediction accuracy. However, we can observe that when precision is reduced below a particular threshold (16 bit fp), the accuracy of the model is greatly affected. Thus, quantization may be performed successfully to conserve energy but quantizing below 16-bit fp may require retraining and fine-tuning to restore the accuracy of the model.

The total energy consumed by an embedded computing application is the sum of the energy required to fetch data from the available memory storage and the energy required to perform the necessary computation in the processor. Table 17 shows the energy required to perform different operations in an ASIC. It can be observed from Table 17 that the amount of energy required to fetch data from the SRAM is much less, than when fetching data from the off-chip DRAM and very minimal if the computation is done at the register files. From this insight, we can conclude that computation should be done as close to the processor as possible to save energy. However, this is a bottleneck because the standard size of available on-chip memory in embedded architectures is very low compared to the size of deep learning models [124]. Algorithmic-based optimization techniques for model compression such as parameter pruning, sparsity, and quantization may be applied to address this challenge [150]. Also, hardware design-based optimizations such as Tiling and data reuse may be utilized [25]. The next section expatiates some of these optimization methods in further detail. Furthermore, most machine-learning models, especially deep learning models, require huge amounts of multiply and accumulate (MAC) operations for effective training and inference. Figure 3 describes the power consumed by the MAC unit as a function of the bit precision adopted by the system. We may observe that the higher the number of bits, the higher the power consumed. Thus, to reduce the power consumed during computation, reduced bit precision arithmetic and data quantization may be utilized [151].

### 4.2. Memory Footprint

The available on-chip and off-chip memory in embedded systems are very limited compared to the size of ML parameters (synapses and activations) [27]. Thus, there is a bottleneck for storing model parameters and activations within this constrained memory. Network pruning (removing redundant parameters) [150] and data quantization [151] (reducing the number of bits used to represent model parameters) are the primary optimization techniques adopted to significantly compress the overall model size such that they can fit into the standard memory sizes of embedded computers.

### 4.3. Latency and Throughput Concerns

Embedded systems are required to meet real-time deadlines. Thus, latency and overall throughput can be a major concern as an inability to meet these tight constraints could sometimes result in devastating consequences. The parameters of deep learning models are very large and are often stored off-chip or in external SDCARDs, which introduces latency concerns. Latency results from the time required to fetch model parameters from off-chip DRAM or external SDCARDs before appropriate computation can be performed on these parameters [150]. Thus, storing the parameters as close as possible to the computation unit using Tiling and data reuse, hardware-oriented direct memory access (DMA) optimization techniques would reduce the latency and thus, inform high computation speed [152]. In addition, because ML models require a high level of parallelism for efficient performance, throughput is a major issue. Memory throughput can be optimized by introducing pipelining [20].

### 4.4. Prediction Accuracy

Although deep learning models are tolerant of low bit precision [153], reducing the bit precision below a certain threshold could significantly affect the prediction accuracy of these models and introduce no little errors, which could be costly for the embedded application. To address the errors which model compression techniques such as reduced precision or quantization introduce, the compressed model can be retrained or fine-tuned to improve precision accuracy [124,150,154,155].

### 4.5. Some Hardware-Oriented and Algorithm-Based Optimization Techniques

Hardware acceleration units may be designed using custom FPGAs or ASICs to inform low latency and high throughput. These designs are such that they may optimize the data access from external memory and/or introduce an efficient pipeline structure using buffers to increase the throughput of the architecture. In sum, some hardware-based optimization techniques are presented in this section to guide computer architects in designing and developing highly efficient acceleration units to inform high performance

#### 4.5.1. Tiling and Data Reuse

Tiling is a technique that involves decomposing a large volume of data into small tiles that can be cached on-chip [25,156]. This technique targets the bottleneck of memory footprint in resource-constrained environments. This technique also introduces scalability to the entire architecture as different sizes of volume data can be easily broken down into bits that may be stored on-chip. Much more, this technique reduces the latency of the system as tiled inputs may easily be reused for computation without having to re-fetch parameters from off-chip. Furthermore, since tiled data can be stored on-chip, energy consumption is reduced. Hardware accelerators may be designed and developed to integrate a tile unit to carry out the tiling process [22]. The pseudocode of the tiling process is given in Algorithm 2.
**Algorithm 2 Pseudocode of the Tiling Process****Require:** Ni: the number of the input neurons No: the number of the output neurons Tile_Size: the tile size of the input data batchsize: the batch size of the input data **for***n* = 0; *n* < *batchsize*; *n* ++ **do** **for**
*k* = 0; *k* < *Ni*; *k*+ = *Tile_Size*
**do** **for**
*j* = 0; *j* < *No*; *j* ++ **do** *y*[*n*][*j*] = 0; **for**
*i* = *k*; *i* < *k* + *Tile_Size*&&*i* < *Ni*; *i* ++ **do** *y*[*n*][*j*] + = *w*[*i*][*j*] * *x*[*n*][*i*] **if**
*i == Ni* − 1 **then** *y*[*n*][*j*] = *f*(*y*[*n*][*j*]); **end if** **end for** **end for** **end for** **end for**

#### 4.5.2. Direct Memory Access and On-Chip Buffers

More recent FPGA architectures owing to the limitation in computation and memory of custom FPGAs, provide general-purpose processors and external memory to offload computation from the FPGA processing logic to the CPU [157]. This architectural organization if not properly utilized, can result in latency concerns. DMAs are units that transfer data between the external memory and the on-chip buffers in the processing logic of the FPGA [152]. Thus, optimizing this process would lead to an efficient performance in execution speed. 

#### 4.5.3. Layer Acceleration

A deep learning network is made up of different kinds of layers (pooling, normalization, fully connected, convolution, etc.). A technique for speeding up the rate of execution of a network and saving memory storage is to design and develop specialized architectures to accelerate particular layers in the network layer [26]. In [22], an accelerator is designed using FPGA technology to accelerate certain parts of a deep neural network. Also, in [51], an accelerator is designed to accelerate convolution layers in a CNN. Network layer acceleration is a hardware-oriented optimization scheme and could pose challenges such as hardware design and high time-to-market since specialized architectures are often considered [148].

#### 4.5.4. Network Pruning

Network pruning is concerned with the removal of certain parts of the network, ranging from weights to layers that do not contribute to the overall efficiency of the network [158]. It entails rounding off certain unused weights and activations to zero to reduce the total memory and computation resource required for efficient computation. These weights and activations are such that they would not alter the accuracy of the execution of the model if avoided. Pruning can either be structured or unstructured [158]. In [124], the pruning technique is employed to compress a neural network within the resource-constrained environments of embedded and mobile devices. The pruning entailed removing i.e., set to zero, weights that are lower than a certain threshold value, and the pruned network is then retrained to improve accuracy.

#### 4.5.5. Reduced Precision

Machine learning algorithms, particularly deep learning models, were originally implemented using a high floating-point number representation format [159,160]. The floating-point number system is made up of a sign, an exponent, and a mantissa [160]. A single floating-point value can be computed using the formula presented in Equation (12). The floating-point number system is, however, power-hungry and thus unsuitable for embedded machine learning applications owing to the resource constraints [161,162]. More so, floating-point arithmetic currently faces drawbacks like manipulating overflow, underflow, and exceptions [163]. This has thus made the fixed-point number system a better alternative owing to the reduced complexity and power consumption of its implementation with the combined range given in Equation (13), [164,165]. Hwang and Sung [166] Investigate the ternary quantization of a Feedforward deep neural network using fixed-point arithmetic. Additionally, [167] considers training a deep network using 16-bit fixed-point arithmetic with stochastic rounding to minimize accuracy loss. Although fixed point arithmetic is power efficient, it is not suitable for representing deep learning parameters that are non-linear. Gustafson and Yonemoto [168] present a new number system called Posit given in Equation (14). Langroudi et al. [163] adopt the posit number system in training a deep convolutional neural network.

(12) $$value={\left(-1\right)}^{sign}\times \left(1+\frac{mantissa}{2^{23}}\right)\times 2^{\left(exponent-127\right)}$$

where *value* is the floating-point value, *sign* is the sign bit, *mantissa* is the mantissa bit.

(13) $$-2^{QI-1}\leq \;\propto \;\leq \left(2^{QI-1}-2^{-QF}\right)|\varepsilon =2^{-QF}$$

where ∝ represents the input integer, *QI* = # of integer bits and *QF* = # of fractional bits and ε is the resolution of the fixed-point number.

(14) $$X={\left(-1\right)}^{sign}\times {\left(useed\right)}^{r_{value}}\times 2^{exponent}\times \left(1+fraction\right)$$

where *X* represents the Posit value, $r_{value}$ represents the number regime and useed represents the scale factor.

#### 4.5.6. Quantization

SVM and DNN model parameters are often represented using 32-bit floating-point values [92,158], which are highly computationally and memory intensive. However, research shows that these models can be implemented efficiently using low precision parameters (8-bit or 16-bit) with minimal accuracy loss [113,169]. Quantization describes techniques aimed at reducing the bit width of the weights and activations of a machine-learning model to reduce the memory storage and communication overhead required for computation. This process thereby reduces the bandwidth required for communication, overall power consumption, area, and circuitry required to implement the design. Many research works have considered different quantization techniques for deep learning models. Courbariaux et al. [170] consider training a deep model using binary representation (+1 and −1), of the model parameters using a binarization function given in Equation (15). Also, [166] proposes a quantization scheme using ternary values (+1, 0, −1). The proposed equation is given in Equations (16) and (17). Other quantization techniques involving Bayesian quantization, weighted entropy-based quantization, vector quantization, and two-bit networks are adopted in [50,51,158], and [171], respectively. Although quantization techniques increase execution speed, the algorithm requires fine-tuning to avoid accuracy loss [172,173]:

(15) $$x^{b}=Sign\;\left(x\right)=\{\begin{array}{ll} +1 & if\;x\geq 0,\\ -1 & Otherwise \end{array}$$

where $x^{b}$ is the binarized variable (weights and activations) and *x* is the real-valued variable.

(16) $$w_{ij,\;new}=w_{ij}-\alpha \left(\frac{\delta E}{\delta w_{ij}}\right)=w_{ij}-\alpha \left(\delta_{i}y_{j}\right)$$

where $w_{ij}$ is the new ternarized weight, α is the learning rate *E* is the output error, $y_{j}$ is the output signal and $\delta_{i}$ is the error signal.

(17) $$\emptyset^{i}\left(x\right)=\emptyset \left(x\right)\left(1-\emptyset \left(x\right)\right)=y\left(1-y\right)$$

where ∅(x) are the new ternarized activation functions and *y* is the output signal.

## 5. Areas of Applications of Intelligent Embedded Systems

Indeed, numerous application areas of embedded systems and the breakthrough of machine learning methods have further widened and deepened the range of applications where embedded systems and machine learning methods are currently actively adopted. Some areas of applications we consider in this survey are intelligent sensor systems and IoTs, deep learning in mobile devices, deep learning training using general-purpose GPUs, deep learning in heterogeneous computing systems, Embedded field programmable gate arrays, energy-efficient hardware design, and architectures.

### 5.1. Intelligent Sensor Systems and IoTs

There is a growing interest revolving around the efficient implementation of machine learning algorithms within embedded environments of sensor networks and the internet. Diverse machine learning algorithms such as SVMs, GMMs, and DNNs are finding useful applications in cogent areas such as the network configuration of mobile networks, analysis of sensor data, power consumption management, etc. A list of applications of machine learning executed within these environments is presented in Table 18. Although machine learning techniques have found useful applications in embedded systems domains, there are major drawbacks that entail limited available computational and memory resources in embedded computing systems.

### 5.2. Deep Learning In Mobile Devices

DNNs are finding very useful applications in mobile devices for speech recognition, computer vision, and natural language processing, respectively, indoor navigation systems, etc. A list of application areas of deep learning models in mobile devices is presented in Table 19. The computational and memory demands required for training and inferencing deep learning models make current mobile devices unsuitable for these models. Thus, more research is being carried out towards the inferencing of the models on mobile devices. There are a lot of energy-intensive applications on current mobile devices which compete for the limited available power and thus, more research is being carried out to optimize these deep learning models so they can efficiently fit within mobile devices. 

### 5.3. Deep Learning Training Using Graphic Processors (GPUs)

DNNs are computationally intensive. State-of-the-art hardware for training deep learning models are graphic processors because of their high-level parallel processing and high floating-point capability. Deep learning algorithms are largely dependent on parallel processing operations, which GPUs are adequately developed to target. Although GPGPUs have very good performance, they are highly power-hungry and expensive to implement, and this thus makes them unsuitable for embedded systems design and development. This owes to the fact that a key design metric for embedded devices is that they must consume very low power and must be economical. Table 20 presents an area of application in training deep learning models on GPGPUs.

### 5.4. Deep Learning Using Heterogeneous Computing Systems

Multicore and many-core architectural enhancement is a modification made by computer architects to address the performance wall and memory wall facing CPU technology. Multicore technology is also called homogenous computing systems, while many core architectures are used in heterogeneous computing systems. Heterogeneous computing systems are systems with more than one type of processor core. Most heterogeneous computing systems are used as acceleration units for offloading computationally intensive operations from the CPU, thereby increasing the system’s overall execution speed. Table 21 presents an area of application of deep learning training in heterogeneous computing systems. A critical drawback in heterogeneous computing systems pivots around the sharing of memory resources, data bus, etc. If designed inefficiently, it can result in data traffic and thus increase latency and power consumption.

### 5.5. Embedded Field Programmable Gate Arrays (FPGAs)

FPGAs are gaining popular interest in the computing world due to their low cost, high performance, energy efficiency, and flexibility. They are often used to design acceleration units and pre-implement ASIC architectures. Table 22 presents certain areas of applications where FPGA architectures are adopted to accelerate deep learning model execution. FPGAs, although programmable and reconfigurable, are difficult to program. This is a critical limitation to their ubiquitous utilization in the embedded computing design.

### 5.6. Energy Efficient Hardware Design and Architectures

The urgency for novel energy-efficient hardware designs cannot be overemphasized. Table 23 presents different application-specific architectures and current research issues involved in targeting the high-performance applications required in this big data era. Application-Specific Architectures, although highly efficient, are difficult to design and implement, having high time-to-market. Their good performance owing to their specificity makes them very suitable for embedded and machine learning applications.

## 6. Research Directions and Open Issues

Embedded machine learning research is still in its early days. Thus, there remains a large range of opportunities to explore in this research direction, which is critical to the development of IoT devices of the future. Some research directions in the areas of Computer Architecture, Deep Learning Optimizations, Hardware Security, Energy Efficiency, and Power Management are presented in Table 24. Additionally, the key lessons learned are highlighted in Section 6.1.

### 6.1. Lessons Learned

In this section, we present a comprehensive summary of the lessons learned from this survey. Our summary covers embedded systems computing architectures, machine learning techniques, deep learning models, optimization techniques, and energy efficiency and power management techniques. 

*Lesson one:* As of today, even the most expensive embedded system platforms do not have the computational and memory capacity to execute expensive machine learning algorithms efficiently. Thus, to bring these models into the embedded space where it becomes a part of our everyday life, which may be found in mobile devices and other IoTs, huge hardware architectural modifications and algorithm optimizations, are required. To approach the issue of overall efficiency properly, optimization approaches ought to tackle the key performance constraints of embedded systems: low power consumption, memory footprint, and latency and throughput concerns. Some optimization techniques are network pruning, data quantization, tiling, layer acceleration, etc.

*Lesson two:* The hardware architectural modifications required to accelerate state-of-the-art deep learning and other machine learning models greatly depend on the ML Model architecture. For example, the CNN model architecture is such that is computation-centric because there are many convolution layers in a CNN architecture, while a fully connected DNN is memory-centric because the algorithm architecture is such that it places great demand on the memory for both storage and throughput. Thus, when hardware acceleration units are being developed, it must be done with a sound understanding of the algorithm architecture to accelerate the right operations, whether vector products (convolutions) or multiply and accumulate (fully connected). This improves the overall efficiency of the hardware architecture. 

*Lesson three:* Most machine learning models are much bigger than standard embedded on-chip and off-chip memory sizes. Thus, to address the memory concern, optimizations may be carried out using network model pruning to reduce the number of parameters, so they can be stored within the available memory, data quantization, which reduces the bit precision of the network parameters so they could fit into standard DRAM and SRAM sizes. Also, direct memory access units may be adopted to reduce the latency of data transfer from the external memory to the processing logic to inform high execution speeds. 

*Lesson four:* To address the energy-efficiency bottleneck, which is primarily due to the type of computation carried out, and the power required to fetch parameters from off-chip memory, optimizations may involve reducing the total number of parameters of the network so that computation may be done as close as possible to the compute unit. Some techniques for compressing the model involve network pruning, clustering, and data quantization. Also, bit reduction using quantization introduces accuracy errors to the overall prediction process. It is worthy of note that precision should not be reduced below a particular threshold which should preserve the model accuracy. To address the accuracy concerns, the quantized parameters of the model may be retrained and fine-tuned to restore prediction confidence.

*Lesson five:* To tackle latency concerns that are a result of off-chip memory transfers, optimizations may be carried out such that model parameters may be cache on-chip for data reuse. This optimization can be done using techniques such as tiling or simple vector decomposition, where input data may be partitioned into bits or tiles that can fit into on-chip memory and may be reused for computation when required. This technique avoids frequent off-chip memory transfers, which is a major concern for both latency and power consumption. Hardware acceleration units may be designed to integrate a Tiling Unit to carry out this operation at the hardware level. Some other techniques to inform high throughput involve pipelining, on-chip buffer optimization, data access optimizations, etc.

*Lesson six:* Although hardware acceleration using custom FPGA logic, GPUs, or CPUs addresses compute power demands, a most promising solution is to develop application-specific architectures using ASICs. Interestingly, every processor architecture has its pros and cons such as the energy efficiency and reconfigurability of FPGAs, but they are slow and hard to program, the high performance of GPU processors but they are power-hungry, the flexibility of general-purpose CPU architectures but are slow with ML computations, etc. Of all these processor architectures, ASICs possess the best performance in terms of energy efficiency because they are hardwired designs to target a specific application. They consume very low power and incur very low costs too. They, however, trade-off flexibility for performance and take a lot of time to market. ASICs are thus gaining renewed interest in the design and development of application-specific machine learning architectures, with Google TPU being a successful case study.

## 7. Conclusions

Machine learning models are fast proliferating embedded devices with limited computational power and memory space. These machine learning models are compute and memory intensive and thus, face the critical limitation of available hardware resources in embedded and mobile devices. In this paper, optimization techniques and various applications of machine learning algorithms within resource-limited environments are presented. We first survey the embedded machine learning space to determine the common machine learning algorithms adopted and select key compute and memory-intensive models such as HMMs, *k*-NNs, SVMs, GMMs, and DNNs. We survey specialized optimization techniques commonly adopted to squeeze these algorithms within resource-limited environments. Also, we present different hardware platforms such as microcontroller units, mobile devices, accelerators, and even TinyML frameworks, which are used to port these algorithms to resource-limited MCUs. Furthermore, we survey the challenges encountered in embedded machine learning and present a more detailed exposition on certain hardware-oriented and algorithm-oriented optimization schemes to address these bottlenecks. Additionally, an exciting look is given to different hardware and algorithm-based optimization techniques, including model pruning, data quantization, reduced precision, tiling, and others to determine which optimization technique best suits the different ML algorithms. Interesting and viable application areas, open research issues, and key take-away lessons are presented in this intersection of embedded systems and machine learning. Conclusively, this survey attempts to create awareness for the passionately interested researcher to kick-start an adventure into this promising landscape of embedded machine learning.

## Figures and Tables

**Table 1:** Machine learning techniques.

Machine Learning Techniques			
Supervised Learning		Unsupervised Learning	Reinforcement Learning
Classification	Regression	Clustering	Genetic Algorithms
SVM	SVR	HMM	Estimated Value Functions
Naïve Bayes	Linear Regression	GMM	Simulated Annealing
*k*-NN	Decision Trees	*k*-means	
Logistic Regression	ANN	DNN	
Discriminant Analysis	Ensemble Methods		
DNN	DNN		

**Table 2:** Machine Learning Techniques in Resource-Constrained Environments.

Reference	ML Method	Embedded/Mobile Platform	Application	Year
[2]	SVM	ARMv7, IBM PPC440	Network Configuration	2015
[20]	DNN	FPGA Zedboard with 2 ARM Cortex Cores	Character Recognition	2015
[22]	DNN	Xilinx FPGA board	Image classification	2016
[23]	LSTM RNN	Zynq 7020 FPGA	Character Prediction	2016
[25]	CNN	VC707 Board with Xilinx FPGA chip	Image Classification	2015
[39]	GMM	Raspberry Pi	Integer processing	2014
[40]	*k*-NN, SVM	Mobile Device	Fingerprinting	2014
[41]	*k*-NN	Mobile Device	Fingerprinting	2014
[42]	*k*-NN, GMM	Mobile Device	Mobile Device Identification	2015
[43]	SVM	Xilinx Virtex 7 XC7VX980 FPGA	Histopathological image classification	2015
[44]	HMM	Nvidia Kepler	Speech Recognition	2015
[45]	Logistic Regression	Smart band	Stress Detection	2015
[46]	*k*-means	Smartphone	Indoor Localization	2015
[47]	Naïve Bayes	AVR ATmega-32	Home Automation	2015
[48]	*k*-NN	Smartphone	Image Recognition	2015
[49]	Decision Tree	Mobile Device	Health Monitoring	2015
[50]	GMM	FRDM-K64F equipped with ARM Cortex-M4F core	IoT sensor data analysis	2016
[51]	CNN	FPGA Xilinx Zynq ZC706 Board	Image Classification	2016
[52]	CNN	Mobile Device	Mobile Sensing	2016
[53]	SVM	Mobile Device	Fingerprinting	2016
[54]	*k*-NN, SVM	Mobile Device	Fingerprinting	2016
[55]	*k*-NN	Xilinx Virtex-6 FPGA	Image Classification	2016
[56]	HMM	Arduino UNO	Disease detection	2016
[57]	Logistic Regression	Wearable Sensor	Stress Detection	2016
[58]	Naïve Bayes	Smartphone	Health Monitoring	2016
[59]	Naïve Bayes	Mobile Devices	Emotion Recognition	2016
[60]	*k*-NN	Smartphone	Data Mining	2016
[61]	HMM	Smartphone Sensors	Activity Recognition	2017
[62]	DNN	Smartphone	Face detection, activity recognition	2017
[63]	CNN	Mobile Device	Image classification	2017
[64]	SVM	Mobile Device	Mobile Device Identification	2017
[65]	SVM	Jetson-TK1	Healthcare	2017
[66]	SVM, Logistic Regression	Arduino UNO	Stress Detection	2017
[67]	Naïve Bayes	Smartphone	Emotion Recognition	2017
[68]	*k*-means	Smartphones	Safe Driving	2017
[69]	HMM	Mobile Device	Health Monitoring	2017
[70]	*k*-NN	Arduino UNO	Image Classification	2017
[71]	SVM	Wearable Device (nRF51822 SoC+BLE)	Battery Life Management	2018
[72]	SVM	Zybo Board with Z-7010 FPSoC	Face Detection	2018
[73]	CNN	Raspberry Pi + Movidus Neural Compute Stick	Vehicular Edge Computing	2018
[74]	CNN	Jetson TX2	Image Classification	2018
[75]	HMM	Smartphone	Healthcare	2018
[76]	*k*-NN	Smartphone	Health Monitoring	2019
[77]	Decision Trees	Arduino UNO	Wound Monitoring	2019
[78]	RNN	ATmega640	Smart Sensors	2019
[79]	SVM, Logistic Regression, *k*-means, CNN	Raspberry Pi	Federated Learning	2019
[80]	DNN	Raspberry Pi	Transient Reduction	2020
[81]	MLP	Embedded SoC (ESP4ML)	Classification	2020
[82]	HMM	Smartphone	Indoor Localization	2020
[83]	*k*-NN	Smartphone	Energy Management	2020
[84]	ANN, Decision Trees	Raspberry Pi	Classification and Regression	2021

**Table 3:** Problems and Algorithms of HMM.

Problem	Algorithm	Definitions	Equation
Likelihood	Forward Algorithm	P(Z|X) is the likelihood, Z is hidden state sequence and X is observation sequence	(1)
Decoding	Viterbi Algorithm	$v_{t}\left(j\right)$ is the Viterbi probability, $v_{t-1}\left(i\right)$ is the previous Viterbi path probability, $a_{ij}$ is transition probability and $b_{j}\left(x_{t}\right)$ is the state observation likelihood	(2)
Learning	Baum-Welch Algorithm or Forward-Backward Algorithm.	*X* is a sequence of observations, *Z* is a hidden state sequence, and λ is the HMM model	(3)

**Table 4:** Optimization Schemes for HMM.

Reference	Optimization Scheme	Application	Comments
[87]	Optimal HMM parameter selection reduced precision.	Speech Synthesis	This technique reduces general computation time and memory footprint however, fixed-point representation introduces accuracy errors to synthesizing.
[88]	HMM parameter reduction, model compression, feature vector size reduction, reduced precision	Speech Synthesis	This research successfully compressed the HMM from 293 MB to 3.61 MB. However, the accuracy of the speech synthesis is just fair owing to huge parameter reduction.

**Table 5:** Important equations involved in *k*-NNs.

Function	Definitions	Equation
*k*-NN Prediction Function	$\hat{y}$ is the predicted output, *x* is input and ρ is Top_1_ and σ is the identity function.	(4)
Euclidean distance	D(p,q) is the Euclidean distance, *p* and *q* are subjects to be compared with n characteristics.	(5)

**Table 6:** Some kNN optimization Schemes.

Reference	Optimization Scheme	Application	Comments
[90]	Binary code learning	Image classification	Although the *k*-NN model is highly compressed, accuracy is traded off for memory efficiency
[55,91]	FPGA Acceleration	Classification	Although computation time is reduced, FPGAs are difficult to program.
[70]	Model compression using Stochastic Gradient Descent	Binary and Multi-class classification	The *k*-NN model is highly compressed with good accuracy

**Table 7:** SVM Optimization Schemes.

TRAINING PHASE OPTIMIZATIONS			
REFERENCE	Optimization Method	Overview	Comments
[98]	Chunking	This method solves the quadratic programming problem (QP) through the removal of rows and columns of zero Lagrange multipliers, thus reducing the entire memory footprint of the SVM model.	Although chunking reduces the size of the model, for large data sets, even the reduced non-zero multipliers are larger than the available memory of the system.
[99]	Decomposition	This method targets replacing the original QP problem with a sequence of smaller problems and, for every sub-problem, invokes an iterative numerical routine, which is expected to converge optimally, thus reducing memory footprint and computation requirement.	At every step, a numerical QP solver which may require numerical precision issues to be addressed is adopted. This process requires invoking an entire iterative QP library routine, thus introducing more computation time.
[96]	Sequential Minimal Optimization	This is the most widely used optimization technique. It involves breaking down the QP problem into sub-problems and subsequently solve the smallest possible optimization problem at every step.	By solving the smallest possible optimization problem at every step, SMO avoids extra matrix memory storage and invoking an entire library at every step. This technique thus saves computation time and memory footprint.
[93]	Accelerated SMO	In this research work, the SMO algorithm is accelerated by introducing an efficient adaptive method for processing data input, incorporating a two-level parallelization scheme, avoiding caching, reducing shrinking, and integrating data reuse.	This technique is not suitable for real-time applications and requires high computational resources to implement successfully.
[100]	Reduced SVM	In this work, a Smoothing Algorithm is proposed to solve the QP problem. The QP problem is first broken down by reducing the variables and constraints of the model. These samples can adequately represent the original variables and constraints of the entire dataset, and subsequently, the proposed algorithm is used to solve the problem.	This technique reduces the computational requirement of SVM but targets only non-linear kernel types.
INFERENCE PHASE OPTIMIZATIONS			
[101]	Fixed Point Optimization	In this research, the SVM algorithm is implemented using fixed-point arithmetic. Fixed-point number representation, when compared with contemporary floating-point, is less compute-intensive. Thus, this reduced precision technique is adopted to execute the SVM model within a smartphone for a multiclass human activity recognition application.	When reducing precision, accuracy becomes a concern because the lower the bit precision adopted, the more likely it is to introduce classification errors.
[102]	Quantization	In this work, a probabilistic technique is used to observe the effect of introducing quantization to the SVM model. The parameters of the model are quantized and using two datasets (Iris and Sonar) which are embedded system-oriented models, the effect of quantization is measured	Although quantization reduces computation time and memory usage, the process introduces noise which could introduce errors except when fine-tuned.
[103]	Logarithmic SVM	In this research, instead of the contemporary floating-point or fixed-point arithmetic, the SVM classification is carried out using the Logarithmic Number System (LNS). The LNS is suitable for low-power applications as it avoids multiplication computations. This saves computation time. The LNS is suitable for machine learning kernel operations which are the computationally intensive sections of the ML algorithm.	Although LNS saves computation time, it requires more hardware resources to implement because it replaces multiplication computations with addition and subtraction.
[104]	Multiplication free SVM	In this research, To address the limitation of hardware resources in resource-constrained environments, The SVM parameters are converted to fixed-point representation, and a hardware-friendly kernel and CORDIC-like iterative algorithm are proposed to avoid multiplication computations using only adders and shifters.	This technique reduces the computational requirements of the system; however, reduced bit precision (fixed-point representation) introduces accuracy errors.
[94]	Order Model Selection	In this research, a model selection algorithm is proposed to select the hyper-parameters with the best Pareto-optimal state using the classification error and the number of support vectors.	This research adopted quantization techniques that introduce classification errors due to the influence of noise.

**Table 8:** Some GMM Optimization Schemes.

Reference	Optimization Method	Application	Comments
[106]	Minimization of Floating-Point Computations	Background Subtraction	The results of this research were impressive, showing no degradation in accuracy except for lower recall rates.
[107]	Comprehensive Sensing	Background Subtraction for real-time tracking in Embedded Vision	The results of this research reveal good performance for computational speed and reduce the memory footprint by 50%
[39]	Integer-based technique	Background/foreground Segmentation	This work shows good performance for processors without FPU, thus reducing computation cost and reducing the memory footprint to 1/12 of the original GMM; however, it cannot be adopted for models with more than two Gaussians

**Table 9:** Description of some DNN models and their parameters.

Name	FC	Conv	Vector	Pool	Total	Nonlinear Function	Weights
MLP0	5				5	ReLU	20 M
MLP1	4				4	ReLU	5 M
LSTM0	24		34		58	Sigmoid, tanh	52 M
LSTM1	37		19		56	Sigmoid, tanh	34 M
CNN0		16			16	ReLU	8 M
CNN1		72		13	89	ReLU	100 M

**Table 10:** Parameters for popular CNN models.

	**CAFFENET** [51]		**ZF**		**VGG11** [51]	
LAYER	Weights	FLOP	Weights	FLOP	Weights	FLOP
CONV1	30 K	210 M	10 K	340 M	0.00	170 M
CONV2	31 K	900 M	610 K	830 M	70 K	1850 M
CONV3	88 K	300 M	880 K	300 M	880 K	5550 M
CONV4	66 K	450 M	1330 K	450 M	3540 K	5550 M
CONV5	44 K	300 M	880 K	300 M	4720 K	1850 M
FC1	37.75 M	80 M	52.43 M	100 M	102.76 M	210 M
FC2	16.78 M	30 M	16.78 M	30 M	16.78 M	30 M
FC3	4.1 M	10 M	4.10 M	10 M	4.1 M	10 M
	**VGG16** [51]		**VGG19** [51]		**ALEXNET**	
LAYER	Weights	FLOP	Weights	FLOP	Weights	FLOP
CONV1	40 K	3870 M	40 K	3870 M	35 K	211 M
CONV2	220 K	5550 M	220 K	5550 M	307 K	448 M
CONV3	1470 K	9250 M	2060 K	12950 M	885 K	299 M
CONV4	5900 K	9250 M	8260 K	12950 M	663 K	224 M
CONV5	7080 K	2310 M	9440 K	3700 M	442 K	150 M
FC1	102.76 M	210 M	102.76 M	210 M	38 M	75 M
FC2	16.78 M	30 M	16.78 M	30 M	17 M	34 M
FC3	4.1 M	10 M	4.10 M	10 M	4 M	8 M

**Table 11:** Some DNN Optimization Schemes.

Ref.	Embedded Architecture	Model	Optimization Techniques	Application
[20]	FPGA(Zedboard development board)	DNN(Mnist)	Data Access Optimization, Time-sharing computation	Image Recognition
[22]	Xilinx XC7Z2020 FPGA	DNN(Mnist)	Tiling and reuse techniques, FIFO Buffering, Pipelining	Image Recognition
[51]	Xilinx Zynq ZC706 FPGA	CNN(VGG16)	Simple Vector Decomposition, Quantization, Tiling, and reuse, Buffer Design	Image Classification
[112]	Altera Stratix-V FPGA (DE5-Net, P395-D8)	CNN(AlexNet, VGG)	Number Precision, Throughput optimization through design space exploration.	Image classification
[25]	Virtex7 VX485T FPGA	CNN	Loop unrolling, Pipelining, Tiling, and data reuse	Image classification
[111]	Xilinx XC6VLX240T FPGA	CNN(LeNet-5), DNN(AlexNet)	Acceleration of Activation function, Pipelining	Image recognition and Classification.
[27]	Xilinx Vertex-7 FPGA	CNN(AlexNet-5)	Network Pruning, Quantization, hardware acceleration	Computer Vision
[125]	Intel Core i7 CPU	CNN(LeNet-5)	Adaptive Quantization	Image classification
[23]	Xilinx Zynq ZC7020 FPGA	RNN(LSTM)	Data Access Optimization, reduced precision, Buffer design	Speech Recognition

**Table 12:** Microcontroller units comparison.

Ref.	Name	Processor	Clock Frequency	Flash	SRAM	Current Consumption (mA)
[128]	Arduino Uno	ATmega328P	16 MHz	32 KB	2 KB	12 mA
[129]	Arduino Mega	ATmega2560	16 MHz	256 KB	8 KB	6 mA
[129]	Arduino Nano	ATmega2560	16 MHz	26-32 KB	1-2 KB	6 mA
[130]	STM32L0	ARM Cortex-M0	32 MHz	192 KB	20 KB	7 mA
[131]	Arduino MKR1000	ARM Cortex-M0	48 MHz	256 KB	32 KB	4 mA
[132]	Arduino Due	ARM Cortex-M3	84 MHz	512 KB	96 KB	50 mA
[133]	STM32F2	ARM Cortex-M3	120 MHz	1 MB	128 KB	21 mA
[134]	STM32F4	ARM Cortex-M4	180 MHz	2 MB	384 KB	50 mA
[135]	RPi A+	ARM Cortex-A7	700 MHz	SD Card	256 MB	80 mA
[135]	RPi Zero	ARMV6	1 GHz	SD Card	512 MB	80 mA
[135]	RPi 3B	ARM Cortex-A53	1.2 GHz	SD Card	1 GB	260 mA
[136]	BeagleV^TM^	RISC-V U74	1.0 GHz	SD Card	8 GB	3000 mA

**Table 13:** Machine learning accelerators.

Accelerator	Processor Type	RAM	Flash Memory	Power	Application	Maker
ARM Ethos NPU	ARM ML processor	1 GB	-	4 TOPs/W	Image processing, voice recognition	ARM
BeagleBone AI	Cortex-A15, Sitara AM5729	1 GB	16 GB	500 mW	Computer vision	Texas Instruments
Google Coral Dev Board	GPU, TPU, CPU	1 GB	8 GB	(6–10) W	Image Processing	Google
Intel Movidus NCS	VPU	1 GB	4 GB	500 mW	Classification, computer vision	Intel
Mustang-F100-A10	Intel Arria 10 GX1150 FPGA	8 GB	-	40 W	Computer vision	Intel
NVIDIA Jetson NANO	GPU, CPU	4 GB 64 it LPDDR4 25.6 GB/s	16 GB eMMC, 5.1 Flash	(5–10) W	Computer vision, audio processing	NVIDIA

**Table 14:** Some literature on mobile machine learning.

Ref.	Ml Algorithm	Optimization Method	Mobile Device	Application
[138]	GoogLeNet	Layer slimming and fine-tuning with model compression,	Samsung Galaxy S5	Computer vision
[139]	GMM	Combination of k-Nearest Neighbor (kNN) and neural networks to improve GMM algorithm.	MIT Mobile device	Speaker Verification
[140]	DNN	Model Compression, reduced precision, and Rescoring Techniques	Nexus 4 Android Phone	Speech recognition
[52]	CNN(Vgg-F, Vgg-M, Vgg-16)	GPU-based acceleration, branch divergence, memory vectorization, and half floating-point computation.	Samsung S5, Samsung Note 4, Samsung S7	Computer Vision
[63]	CNN	Pointwise group convolution and channel shuffling	ARM-based Mobile Device	Image classification, object detection

**Table 15:** Comparison of CloudML and TinyML Platforms.

Computing Technology	Platform	Architecture	Memory	Storage	Power	Ref.
CloudML	Nvidia V100	GPU Nvidia Volta^TM^	16 GB	^1^ TBs-PBs	250 W	[144]
	Nvidia Titan RTX	GPU Nvidia Turing^TM^	24 GB	^1^ TBs-PBs	280 W	[145]
	Nvidia V100S	GPU Nvidia Volta^TM^	32 GB	^1^ TBs-PBs	250 W	[144]
TinyML	ST F446RE	Arm M4	128 KB	0.5 MB	0.1 W	[146]
	ST F746ZG	Arm M7	320 KB	1 MB	0.3 W	[147]
	ST F767ZI	Arm M7	512 KB	2 MB	0.3 W	[147]

^1^ Terabytes to Petabytes.

**Table 16:** TinyML Framework.

Framework	Supported mL Algorithm	Compatible Platforms	Languages	Main Developer
TensorFlow Lite	Neural networks	ARM Cortex-M	C++11	Google
ELL	Neural networks	ARM Cortex-A Raspberry Pi Arduino micro:bit	C C++	Microsoft
ARM-NN	Neural networks	ARM Cortex-A ARM Mali Graphics Processors ARM Ethos Processor ARM Cortex-M	C	ARM
CMSIS-NN	Neural networks	ARM Cortex-M	C99	ARM
STM 32Cube. Al	Neural networks	STM32	C	STMicroelectronics
AlfES	Neural networks	Window [DLL] Raspberry Pi Arduino ATMega32U4 STM32 F4 Series ARM Cortex-M4	C	Fraunhofer IMS
NanoEdge Al Studio	Unsupervised learning	ARM Cortex-M	C	Cartesian
MicroMLGen	SVM RVM	Arduino ESP32 ESP8266	C	Particular developer
Sklearnporter	SVM Decision tree Random Forest Ada BoostClassifier K-NN Neural networks	Multiple constrained & non-constrained platforms	C, GO Java, JavaScript, PHP, Ruby	Particular developer
m2cgen	Linear regression Logistic regression Neural networks SVM Decision tree Random Forest LGBM classifier	Multiple constrained & non-constrained platforms	C, C#, Dart Go, Java JavaScript PHP, PowerShell, Python, R Visual Basic	Particular developer
Weka-porter	Decision trees	Multiple constrained & non-constrained platforms	C, Java JavaScript	Particular developer
EmbML	Decision trees Neural networks SVM	Arduino Teensy	C++	Research group
emlearn	Decision trees Neural networks Naïve Gaussian Bayes Random forest	AVR ATmega ESP8266 Linux	C	Particular developer
uTensor	Neural networks	mBed boards	C++11	Particular developer
TinyMLgen	Neural networks	ARM Cortex-M ESP32	C	Particular developer

**Table 17:** Energy Consumption in (pJ) of performing operations.

Operation	Energy (pJ)
8 bit int ADD	0.03
16 bit int ADD	0.05
32 bit int ADD	0.1
16 bit float ADD	0.4
32 bit float ADD	0.9
8 bit MULT	0.2
32 bit MULT	3.1
16 bit float MULT	1.1
32 bit float MULT	3.7
32 bit SRAM READ	5.0
32 bit DRAM READ	640

Source: Bill Dally, Cadence Embedded Neural Network Summit, 1 February 2017.

**Table 18:** Intelligent sensor systems and IoTs.

Year	Ref.	Application Area	Highlights	Key Findings	Limitations
2015	[2]	Mobile Adhoc Networks (MANETs)	This research investigates the automatic configuration of the radio and IP stack of a MANET at runtime using machine learning techniques. To achieve this, the SVM algorithm was implemented within two communication controllers with general-purpose processors (the ARMv7 and IBM PPC440GX).	To deploy the SVM efficiently, certain optimizations were carried out on the algorithm, and the corresponding effect of each optimization technique was observed whilst comparing each ablation with a baseline. The result of the proposed system showed improved performance reducing the runtime of the system in most cases.	In this research, accuracy was traded off for execution speed. The accuracy of SVM algorithms depends on their floating-point computations. However, floating-point operations were avoided by the optimization scheme, thereby reducing the overall accuracy. Also, computationally intensive machine learning methods are often executed using hardware acceleration units. This increases the accuracy and execution speed of the scheme.
2016	[50]	Intelligent Sensor Networks and the Internet of things	This research work investigates the use of a machine learning model deployed in an embedded environment to analyze sensor data at run time. The research explored the use of a Gaussian mixture model (GMM) on an FRDM-K64F embedded board to achieve this. For retrieving raw data intelligently, the research employed the NXP intelligent sensor framework (ISF), which collects the raw sensor data and stores them for sequel purposes. The GMM is then used to analyze the huge amount of sensor data for classification and clustering to detect the required processes. The intelligent sensor system is used to monitor the conditions of an intelligent stove and a water circulation system.	The GMM algorithm is optimized using the expectation-maximization (EM) algorithm and implemented using the minimum description length (MDL) criterion due to the intensive computational vector and matrix operations required and the limited computational power available. Furthermore, the fixed point number representation was used in the computation of the sensor data, while the hardware implementation was done using the imperfect floating-point unit.	The number representation adopted improves the speed of the implementation at the expense of accuracy. Although the analysis of raw sensor data reduces the network data traffic and enhances data security, employing the use of model parameters to depict sensor data reduces the effectiveness of the GMM since the accuracy of machine learning models largely depends on the volume of data for appropriate training.
2017	[62]	Intelligent Ultra-Low-Power IoT Systems	This research considers the efficient implementation of deep neural networks across diverse low-power IoT computing platforms. The different IoT applications considered in this research cut across voice, image, and activity recognition. The work proposes a fully-connected deep neural network for training the different datasets and consequently inferencing the trained model using an SoC-based hardware accelerator. A unique FC-NN topology is modeled for each application for high performance and trained using preprocessed datasets as required by the application.	The NN model is manually mapped to the hardware accelerator by extracting the weights and biases and converting them from 32-bit floating-point to 16-bit fixed-point values owing to the accelerator constraints indicated in the paper. The quantization method introduced to reduce the precision of the model reduces the prediction accuracy of the system which parameter retraining would address. The performance of this model was measured using accuracy, harmonic mean score, weighted harmonic mean score, and power consumption. The results of the research show that deep learning models can be executed efficiently using specialized hardware processors	Model scalability and battery life performance which are major concerns in IoT developments are not considered in the research.
2018	[71]	Intelligent Wearable Systems	This research investigates the integration of a machine learning algorithm executed on a wearable device to extract, analyze and classify sensor data on the embedded device, thereby saving the energy required to send for centralized processing. To achieve this, the research work entailed (1) sampling the data generated to avoid the generation of redundant input data, (2) carrying out appropriate feature extraction using the Integral of the modulus of acceleration (IMA) embedded machine learning method, which avoids sending the raw sensor data to a centralized processor for knowledge extraction and (3) executing the classification process using support vector machines (SVM). This machine learning sensing system was employed for the energy-efficient long-term monitoring of the physical activities of house occupants.	The results obtained were fascinating, revealing that employing adequate machine learning methods extends the battery life of a wearable sensor by 987 days when compared to transferring raw sensor data through the network. The research makes use of an SoC-based wearable sensor for data collection.	Data sampling reduces system accuracy because machine learning models depend on large sensor data for effective training. Also, SoCs are highly energy-efficient and demonstrate good performance, and they require a very high time-to-market compared to other embedded architectures.

**Table 19:** Deep learning in mobile devices.

Year	Reference	Application Area	Highlights	Key Findings	Limitations
2018	[74]	Image Processing	This research investigates an adaptive machine learning approach to selecting the most optimal DNN for a given input, thereby saving inference time. To achieve this, the research work trained an offline predictor (premodel) using the K-nearest neighbor classification models on 12 DNN models measuring the predictions under two metrics—response to the input data and precision required. The inference time for a DNN model is a function of these two parameters.	The premodel is then executed on embedded hardware to select from an option of 14 pre-trained CNN models, the most optimal model to employ for a particular input data and precision requirement, thereby reducing the inference time. The ML system was implemented on the NVIDIA Jetson TX2 deep learning platform, which had an embedded GPU	GPUs provide the necessary speed and efficiency for deep learning implementations but are power-hungry, making them unsuitable for embedded applications.
2018	[138]	Mobile Computing	This work investigates a novel optimization method for accelerating deep neural network models for resource-constrained environments like mobile devices. The research exploits the observation that deep learning execution time is majorly slowed by the non-tensor layers in the model (pooling, LRN, and normalization layers). An optimization framework, “DeepRebirth,” is proposed, which targets the regeneration of the non-tensor layers in the model using either streamline or branch slimming methods. The optimization method involves merging non-tensor layers with bottom tensor units and further fine-tuning the regenerated layer in streamline streaming. Branch slimming involves merging non-tensor layers with tensor units at the same level.	The performance of the optimized model was measured over the state of the art networks like GoogLeNet, AlexNet, and ResNet on different high-end and low-end mobile computing processors. The results of DeepRebirth reveal that the optimization method yields faster execution time, minimal accuracy loss, and reduced power consumption making the technique appropriate for heterogeneous mobile devices.	It is, however, observed that the optimization technique has little influence on mainstream ARM CPUs, which are the most ubiquitous processors in mobile devices. This limits the impact of optimization in the mobile computing world. More so, the inference was carried out during airplane mode, and thus, the metric for power consumption on mobile devices is not accurately measured. This metric is very important because battery life is a great concern in mobile computing and can constitute a major challenge to deep learning inferencing on mobile computing platforms.
2018	[174]	Indoor Navigation	A framework is presented in this work to improve WiFi-based fingerprinting indoor localization using commodity smart mobile devices. The research proposes the utilization of a CNN model in predicting the location of a user in an indoor environment. The WiFi access points are used in creating the image database, which models the different locations on the navigation path, which are then used to train the CNN model. The real-time AP data are subsequently used by CNN to predict the location of the user at run time.	The CNN model used in the research “CNN-LOC” eliminates the pooling layer owing to the size of the grids, thereby eliminating the need for subsampling. This consequently reduces the training and inference times and power consumption during execution, making it suitable for resource-constrained mobile computing. The research also introduces scalability by proposing a hierarchal classifier for large area localization. The performance of this research is measured by comparing the prediction accuracy across other indoor localization frameworks using SVR, KNN, and DNN approaches. The results show that the CNN-LOC framework outperforms the others.	However, the research overlooks the limitation of battery life in smartphones during the execution of computationally intensive deep learning models like CNN using WiFi APs. WiFi has been observed to be one of the applications responsible for draining the power in mobile devices, thereby shortening the battery life.
2018	[163]	Energy Efficiency of Embedded Devices	This work investigates a novel low precision arithmetic approach to optimize the power consumption and memory utilization of DCNN inferencing in the embedded system domain. To achieve this, the work explored the use of the posit number system over the contemporary fixed and floating-point number representation. The process involves converting the posit number to a decimal floating-point number and subsequently a binary floating-point number for reading and writing to memory and vice versa during the processing period.	The system is executed using three datasets and compared to a reference point which is the single floating-point representation. The results are fascinating in terms of memory utilization during inferencing, and the accuracy obtained despite using low precision estimation	The hardware specifications are not given in the research.

**Table 20:** Deep learning training in general purpose graphic processing units (GPGPUs).

Year	Reference	Application Area	Highlights	Key Findings	Limitations
2019	[19]	GPGPU based CNN Training	GPUs are one of the major hardware acceleration tools for Deep learning algorithms owing to their suitability for high thread-level parallelism. This research Investigates an optimal system configuration for GPGPUs in training CNN models effectively. Two image classification CNN models (LeNet and MiniNet) are executed using different GPU configurations to determine the most optimal configuration. The research employs GPGPU-sim, a GPU simulator, in executing the CNN model whilst observing the effect of modifying the configuration of the GPU in terms of the NoC architecture used, the size of the L1D and the L2D caches, and network traffic intensity on the different layers of the CNNs (convolutional, pooling and fully connected layers).	The result of the research reveals that the Mesh Network outperforms the Perfect Network at the fully connected layers. Also, modification in the size of the L1 affects the performance of the CNN, while changes in the L2 cache do not influence the CNN performance. Also, the network traffic intensity differs across the layers.	GPGPUs are very cost-intensive and energy-intensive. Thus, they are unsuitable for embedded applications.

**Table 21:** Deep Learning in heterogeneous computing systems.

Year	Reference	Application Area	Highlights	Key Findings	Limitations
2015	[16]	Heterogeneous multicore Architecture	A Heterogeneous multi-core architecture to facilitate the high-performance execution of Machine learning algorithms is explored in this research work. The middleware platform called HeteroSpark involves the integration of a GPU accelerator into an already existing CPU-based architecture (Spark) to accelerate computationally intensive machine learning methods. Employing Java virtual machines, data is moved between the CPU and the GPU when required for intensive computations. The GPU is positioned in Spark’s worker nodes. The GPU relieves the CPU of workload, thus accelerating the entire computation process due to GPUs’ thread-level parallelism. The process is abstracted from the user through user-friendly APIs.	The performance of the proposed heterogeneous framework is compared to a baseline (Spark), and the results were fascinating, with GPU acceleration yielding 18.6x when HeteroSpark is scaled up to “32CPU cores: 8 GPUs” and Spark uses “32 Cores: no GPUs”.	The research focuses on a software approach to the problem and not a hardware perspective.

**Table 22:** Embedded FPGAs: optimization and throughput.

Year	Reference	Application Area	Highlights	Key Findings	Limitations
2015	[25]	CNN Optimization using FPGAs	This work proposes a development scheme to accelerate the execution of convolutional neural networks in resource-constrained FPGAs by exploring the design space of the system. The roofline model, which uses a computation and communication approach to measure the performance of the system, was adopted to explore the design space to affect the optimization of the computation and memory access process.	The computation process is accelerated by loop unrolling, pipelining, and tile sizing using a polyhedral-based optimization technique. Memory access is optimized by adopting loop promotion and transformations for efficient data reuse. Optimal parameters are first determined for single convolutional layers and then unified unroll factors are used for CNNs with multiple convolutional layers. The performance of the proposed architecture is estimated by observing the computational performance, resource utilization, and power consumption. The proposed scheme reveals brilliant results in outperforming prior works.	The research, however, focused on accelerating the feedforward inference process and optimized only the convolutional layers neglecting the pooling, normalization, and fully connected layers which are required in real-world scenarios. Besides, tiling introduces some accuracy loss to the model execution.
2015	[20]	Deep Learning FPGA Architecture	The acceleration of deep learning inference, particularly to large-scale networks using an FPGA, is considered in this work. The research exploits the high performance, reduced power consumption, and low-cost advantages of employing the FPGA to accelerate the prediction process of a DNN model. The research was limited to the prediction process. The Accelerator Architecture proposed by the research contained a direct memory access module, a deep learning module with an ARM Cortex CPU. To tackle the challenge of mapping in large neural networks owing to constrained computational resources, a time-sharing computational technique is adopted in the execution of data fragments that have been previously partitioned using the tiling technique.	The performance of the architecture is improved by cache reuse effected by introducing a Block RAM module. Furthermore, the throughput was increased by incorporating a pipelining methodology in the DL module. To address the flexibility challenge of the FPGAs, a software library is proposed to make the system user-accessible. The performance of the proposed model is measured by comparing the results with a MATLAB-based Core2 CPU baseline. The results show improved performance in power consumption and data throughput	The tiling technique introduces some accuracy errors in computation. The research adopted a high floating-point computation. However, a low fixed-point precision approximation of the DNN model if introduced, could further save memory usage and improve performance.
2016	[22]	Energy Efficient Hardware Acceleration	This work targets an FPGA-based accelerator design to improve the training and inference of computationally intensive deep learning models. The research proposes an architecture, “DLAU”, pivoted on introducing scalability to accommodate diverse network sizes of deep learning models and employing the tiling technique, which entails splitting the large volume of data introduced into smaller grids and distributing them to effect the parallel computing technique, which is suitable for deep learning models. The DLAU Acceleration model exploits the computational methods required by deep learning models (matrix multiplication and activation functions).	The architecture introduces three fully pipelined processing units (Tiled Matrix Multiplication Unit, Part Sum Accumulation Unit, and Activation Function Acceleration Unit) to execute these kinds of computations efficiently, thereby offloading the main processor of intensive computations increasing overall execution speed and throughput at low cost. The performance and cost of the proposed model are measured using the execution speed, resource utilization and power consumed. The results reveal that the DLAU accelerator outperforms other acceleration techniques.	The research focuses on hardware design alone. However, FPGAs, although flexible owing to their reconfigurable characteristic, are difficult to program employing a hardware/software approach that is more suitable for developers. More so, power consumption can be further reduced by employing low precision approximation techniques for deep learning models which are not employed in the paper.
2016	[51]	Embedded FPGA	A hardware/software co-design approach to accelerate large-scale convolutional neural networks in the embedded system domain is considered in this work. The framework proposed entails the decomposition and quantization of large-scale CNN models at the software level and the design of an FPGA-based CNN accelerator at the hardware level. The optimization at the software level targets the high memory space required by fully-connected layers and high computational demand required by the convolutional layers in the CNN model, while the hardware level optimization entailed the design of an application-specific FPGA architecture to meet the computational and high memory bandwidth required for the efficient execution of the model. To achieve this, the Singular Value Decomposition (SVD) technique was adopted to accelerate the fully connected layers, and dynamic-precision quantization using 16-bit fixed-point representation was used for the quantization process.	The hardware implementation entailed the design of a heterogeneous architecture of general-purpose processors and an FPGA consisting of appropriate buffers, processors, and memories to effect efficient parallel processing, matrix multiplication, tiling, and data reuse. The performance metrics of the proposed model are evaluated by implanting a very deep VGG CNN and the results are compared to CPU and GPU-based architectures and other FPGA-based CNN accelerators demonstrating brilliant performance.	The research, however, considers the optimization of convolutional and fully connected layers neglecting pooling and normalization layers. Besides, tiling and quantization techniques introduce accuracy errors which fine-tuning techniques ought to address but Parameter fine-tuning is not considered in this literature. Moreover, the reconfigurable advantage of FPGAs is not exploited in the research. Also, embedded platforms face battery life constraints which is also not considered in this research.
2016	[112]	OpenCL-based FPGA Hardware Acceleration	This work considers the acceleration of large-scale convolutional neural networks using an OpenCL-based FPGA Architecture focusing on optimizing the rate at which data is processed to improve throughput. To achieve high throughput, a precision study is carried out using the Caffe tool of two CNN models (AlexNet and VGG) for the convolution and fully connected layers to determine the optimal precision at which inference can be carried to avoid accuracy loss. At the hardware level, scalable modules are designed using OpenCL software design skit for accelerating each of the different layers in the CNN (convolution, pooling, normalization, and fully connected layers). In the OpenCL implementation phase, the convolution layer is accelerated by developing a scalable convolution block that performs the multiply and accumulates operations required by the convolution layer. The Normalization layer is accelerated by developing a module that performs exponent operations using a piece-wise linear approximation function. The pooling and fully connected layers are accelerated by implementing single work-item kernels.	For appropriate hardware resource utilization on the FPGA, a design space exploration is carried out using a genetic algorithm in Matlab to affect throughput. The proposed architecture is used to accelerate the AlexNet and VGG CNN models on two FPGA boards. The results are compared with prior FPGA-based architectures outperforming them.	The research, however, focused on optimizing throughput and not memory utilization which is a key concern for embedded platforms. Besides, power consumption is a key concern owing to the limited battery of embedded computing devices.
2016	[23]	FPGA based Hardware Acceleration	Hardware architecture for the efficient execution of recurrent neural networks (RNN) based on a long short term memory design approach is proposed in this work. The proposed solution targets the constraint involving vanishing or exploding gradients while executing RNNs by recommending a variation to the LSTM design approach. The recommended LSTM design method entails the definition of three gates (input, forget, and output) that learn to either remember, forget or output a result. This LSTM architecture is implemented using the FPGA which uses fixed-point approximations for computations. The matrix-vector multiplications are implemented using Multiply Accumulate units while the non-linear functions are computed using a MAC unit and a comparator. Direct memory access units are incorporated with a stream synchronization module to foster efficient communication of data between the processing logic units and the external memory. The module integrates a driver software to incorporate data reuse to avoid constant external memory accesses which consumes power.	The proposed architecture is implemented using Zedboard’s FPGA with dual ARM Cortex-A9 processor and compared to platforms using CPU and GPU computing units for performance evaluation outperforming them.	However, fixed-point representations introduce accuracy errors to the results, which parameter retraining or model fine-tuning ought to address. Besides, external memory accesses consume a lot of power owing to the high bandwidth requirement. Furthermore, the research focuses on the learning phase alone, neglecting inferencing.

**Table 23:** Energy-efficient hardware design and architectures.

Year	Ref.	Application Area	Highlights	Key Findings	Limitations
2016	[175]	Layer based Hardware Acceleration	This work explores the design of specialized hardware architecture to accelerate the execution of Softmax layers in deep learning applications. The research targets mitigating the challenge of employing existing direct-mapping techniques used for accelerating the hardware implementation of other layers (convolution, pooling, and normalization) of the CNN for the Softmax layer. This challenge pivots on the division and overflow problem which introduces high latency and high accuracy errors owing to the hardware complexity and resource constraints, respectively.	To overcome the division problem, a domain transformation which involves the replacement of the complex division operation with a logarithmic subtraction operation. This reduces the complexity of hardware implementation. Also, the overflow problem is overcome, downscaling is employed to reduce the bit width of the parameters. The downscaling method also reduces the complexity of the logarithmic operations addressing the issue of path delay, which informs reduced latency.	The research considers the hardware orientation of the acceleration process, neglecting the software which is sometimes required for data reuse. Besides, the research only considers the optimization of the Softmax layer; however, the generic optimization involving other layers is not considered.
2017	[29]	Weight/Activation based Hardware Acceleration	This research Investigates a hardware architecture aimed at accelerating the execution of convolution layers in a CNN by exploiting the zero weights and activations. By omitting the multiplication and accumulation of computations containing zero weights and activations often caused by pruning techniques and much more the effect of using the ReLU activation function, the research targets power reduction and faster speed in the execution of CNNs. Unlike other zero-aware accelerator architectures, to exploit both zero weights and activations, each of the Processing Elements (PE) of the proposed architecture performs single convolutions to avoid synchronicity. The proposed architecture contains two on-chip SRAMs (activation and weight), a PE Array, and a ReLU module.	The research implemented two architectures employing two data widths (16 bit fixed point and 5-bit logarithmic quantization). The proposed architecture introduces a challenge involving latency termed load imbalance which a zero-aware kernel allocation approach was used to resolve. The results of the research outperformed other non-zero acceleration architectures and partially zero-aware acceleration architectures.	However, the research only considered the acceleration of convolution layers. On-chip SRAM may be ineffective for large-scale CNNs. Besides, reduced precision and quantization techniques introduce accuracy errors for large-scale networks.
2018	[111]	Activation Function based Hardware Acceleration	A hardware architecture is designed and implemented to accelerate the execution of the activation function in deep neural networks in this research work. The proposed architecture introduces the flexibility of selecting between four activation functions (Sigmoid, Tanh, ReLU, and Softmax function) using a time multiplexing approach. The architecture employs 16-bit fixed-point precision arithmetic for computation and bus width. Furthermore, the piecewise linear interpolation method is employed in the approximate calculation of the activation function. The approximation is carried out using an on-chip lookup table designed using ROM. An address generator module is used to map the neuron data to the Lookup Table using an address mapping algorithm.	The hardware implementation is carried out using a CPU and GPU for training and an FPGA for inference the proposed architecture is used in implementing two CNNs (LeNets-5 and AlexNet) with the results compared using Matlab. The results of this architecture reveal high computation efficiency and flexibility.	However, the architecture is modeled for small neural networks. The on-chip lookup table implemented increases the on-chip memory storage required thus unsuitable for embedded applications with hardware resource constrain.
2020	[13]	Computer Architecture and Chip Design	This research surveys the influence and consequence of deep learning advances in computer architecture and chip design. The research survey points out the requirements of a specialized hardware architecture for deep learning implementation owing to the peculiar mathematical methods used in deep learning computations. The current optimization trends in general-purpose CPUs are not appropriate for these mathematical models more so as Moore’s law has slowed down in recent times.	The research survey suggests low precision approximations for machine learning models to enhance their execution and reduce their power consumption. Furthermore, the research survey reveals how machine learning techniques could also be used in the exploration of the design space for ASIC development.	The research survey focused on the hardware perspective alone. However, there are current software and hardware/software co-design approaches to enhance deep learning. More so, Photonics is a current promising research thrust owing to the promising characteristics of photonics-based architecture over electronic-based architecture.

**Table 24:** Future research directions.

S/N	Key Areas for Future Research	Possible Research Focus
1	Computer Architecture	With the end of Dennard’s scaling and Moore’s law significantly slowing down, combined with the performance wall of Silicon, there remains an urgent need for innovation in the design and development of hardware architecture to meet the target for high-performance computing that face us today. In this respect, [176] suggests domain-specific architectures in the form of GPUs, FPGAs, TPUs, etc. as a research direction for future high-performance architectures. DSAs are different from ASICs in that DSAs introduce flexibility to the design due to their programmability. This is an open research area in computer architecture. Also, to generate highly efficient application-specific ASIC architecture designs, the design space is required to be explored efficiently, which is currently a stringent task carried out by human experts. However, [177,178] presents a frontier of employing machine learning techniques in exploring the design space at design time and runtime. Ruben et al. [179] proposed a machine learning-based prediction for the dynamic, runtime, and architectural optimizations of Embedded Systems. This is another open area of research—adopting machine learning in efficient computer architecture design and development
2	Deep Learning Optimizations	Deep learning models are computationally and memory intensive, and thus, implementing them within resource-constrained environments is tasking. There is, therefore, an opportunity for highly efficient optimization techniques to compress deep learning models efficiently with minimal or no accuracy loss. Although many research works have explored optimization techniques, optimization methods are infinite, and thus there remains an opportunity to optimize deep learning models still. Some optimization techniques are but are not limited to pruning, clustering, layer acceleration, quantization, and numeric precision. Some optimizations combine one or more of these techniques to compress a model successfully.
3	Hardware Security	Software security has been greatly explored, but little work has been done in hardware security which is a major concern in embedded systems development [180]. State-of-the-art embedded hardware architectures are prone to Trojan attacks, and thus, this creates the need for research in the design and development of secure embedded architectures for embedded applications.
4	Energy Efficiency and Power Management	Energy Efficiency is a critical issue in embedded computing systems because most embedded devices run on a battery [181]. Thus, to effect the continuous functionality of embedded devices, there remains the need to adequately design energy-efficient architectures and also adopt innovative power management techniques. This is a crucial research thrust in embedded computing technology, particularly to meet the requirements of high-performance machine learning applications [182,183,184].
5	Silicon Photonics	Current Silicon technology is reaching its limit for performance and thus [185] surveys the exploration of photonics-based architectures as a substitute for silicon technology owing to the high bandwidth and data transfer speed of photonics-based architectures [186,187]. This is a key research direction for high-performance architectures

## Data Availability

Data sharing does not apply to this article.

## Abbreviations

**Abbreviation**	**Full Meaning**
ANN	Artificial Neural Network
ASIC	Application Specific Integrated Circuit
ASIP	Application-Specific Instruction-set Processor
CPU	Central Processing Unit
CNN	Convolutional Neural Network
DCNN	Deep Convolutional Neural Network
DMA	Direct Memory Access
DNN	Deep Neural Network
DSA	Domain-Specific Architectures
DSP	Digital Signal Processor
EML	Embedded Machine Learning
FPAA	Field Programmable Analog Array
FPGA	Field Programmable Gate Array
FC	Fully Connected
GPGPU	General Purpose Graphic Processing Unit
GMM	Gaussian Mixture Model
HMM	Hidden Markov Model
IC	Integrated Circuit
I/O	Input/output
ISA	Instruction Set Architecture
ISF	Intelligent Sensor Framework
*k*-NN	*k*-Nearest Neighbors
LRN	Local Response Normalization
LSTM	Long Short Term Memory
IoT	Internet of Things
MANET	Mobile Adhoc Network
MCU	Microcontroller Unit
PE	Processing Element
RAM	Random Access Memory
RNN	Recurrent Neural Network
SoC	System on Chip
SVM	Support Vector Machines
SDG	Stochastic Descent Gradient
SVD	Singular Value Decomposition
TPU	Tensor Processing Unit
WSN	Wireless Sensor Network

## References

[1] Wayne W. (2007). Praise of High-Performance Embedded Computing: Architectures, Applications, and Methodologies.

[2] Haigh K.Z., Mackay A.M., Cook M.R., Lin L.G. (2015). Machine Learning for Embedded Systems: A Case Study.

[3] Krizhevsky A., Sutskever I., Hinton G. (2012). ImageNet classification with deep convolutional neural networks Alex. Adv. Neural Inf. Process. Syst..

[4] Szegedy C., Liu W., Jia P.Y., Reed S.S., Anguelov D., Erhan D., Vanhoucke V., Rabinovich A. Going deeper with convolutions. Proceedings of the 2015 IEEE Conference on Computer Vision and Pattern Recognition (CVPR).

[5] He K., Zhang X., Ren S., Sun J. Deep residual learning for image recognition. Proceedings of the IEEE Computer Society Conference Computer Vision Pattern Recognition.

[6] Real E., Moore S., Selle A., Saxena S., Suematsu Y.L., Tan J., Le Q.V., Kurakin A. Large-scale evolution of image classifiers. Proceedings of the 34th International Conference Machine Learning ICML.

[7] Tan M., Le Q.V. EfficientNet: Rethinking model scaling for convolutional neural networks. Proceedings of the 36th International Conference Machine Learning ICML 2019.

[8] Hinton G., Deng L., Yu D., Dahl G.E., Mohamed A.R., Jaitly N., Senior A., Vanhoucke V., Nguyen P., Sainath T.N. (2012). Deep neural networks for acoustic modeling in speech recognition. IEEE Signal. Process. Mag..

[9] Chan W., Jaitly N., Le Q.V., Vinyals O. Listen, attend and spell. Proceedings of the 2016 IEEE International Conference on Acoustics, Speech and Signal Processing (ICASSP).

[10] Wu Y., Schuster M., Chen Z., Le Q.V., Norouzi M., Macherey W., Krikun M., Cao Y., Gao Q., Macherey K. (2016). Google’s neural machine translation system: Bridging the Gap between human and machine translation. arXiv.

[11] Collobert R., Weston J., Bottou L., Karlen M., Kavukcuoglu K., Kuksa P. (2011). Natural language processing (almost) from scratch. J. Mach. Learn. Res..

[12] Haj R.B., Orfanidis C. A discreet wearable long-range emergency system based on embedded machine learning. Proceedings of the 2021 IEEE International Conference on Pervasive Computing and Communications Workshops (PerCom Workshops).

[13] Dean J. The deep learning revolution and its implications for computer architecture and chip design. Proceedings of the 2020 IEEE International Solid-State Circuits Conference-(ISSCC).

[14] Cui X., Liu H., Fan M., Ai B., Ma D., Yang F. (2020). Seafloor habitat mapping using multibeam bathymetric and backscatter intensity multi-features SVM classification framework. Appl. Acoust..

[15] Khan M.A., Kim J. (2020). Toward developing efficient Conv-AE-based intrusion detection system using heterogeneous dataset. Electronics.

[16] Li P., Luo Y., Zhang N., Cao Y. HeteroSpark: A heterogeneous CPU/GPU spark platform for machine learning algorithms. Proceedings of the 2015 IEEE International Conference Networking, Architecture Storage, NAS.

[17] Raparti V.Y., Pasricha S. (2018). RAPID: Memory-aware NoC for latency optimized GPGPU architectures. IEEE Trans. Multi-Scale Comput. Syst..

[18] Cheng X., Zhao Y., Robaei M., Jiang B., Zhao H., Fang J. (2019). A low-cost and energy-efficient noc architecture for GPGPUs. J. Nat. Gas Geosci..

[19] Zhang L., Cheng X., Zhao H., Mohanty S.P., Fang J. Exploration of system configuration in effective training of CNNs on GPGPUs. Proceedings of the 2019 IEEE International Conferece Consumer Electronics ICCE.

[20] Yu Q., Wang C., Ma X., Li X., Zhou X. A deep learning prediction process accelerator based FPGA. Proceedings of the 2015 IEEE/ACM 15th International Symposium Cluster Cloud, Grid Computer CCGrid 2015.

[21] Noronha D.H., Zhao R., Goeders J., Luk W., Wilton S.J.E. On-chip FPGA debug instrumentation for machine learning applications. Proceedings of the 2019 ACM/SIGDA International Symposium on Field-Programmable Gate Arrays.

[22] Wang C., Gong L., Yu Q., Li X., Xie Y., Zhou X. (2016). DLAU: A scalable deep learning accelerator unit on FPGA. IEEE Trans. Comput. Des. Integr. Circuits Syst..

[23] Chang A.X.M., Martini B., Culurciello E. Recurrent Neural Networks Hardware Implementationon FPGA. http://arxiv.org/abs/1511.05552.

[24] Branco S., Ferreira A.G., Cabral J. (2019). Machine learning in resource-scarce embedded systems, FPGAs, and end-devices: A survey. Electronics.

[25] Zhang C., Li P., Sun G., Guan Y., Xiao B., Cong J. Optimizing FPGA-based accelerator design for deep convolutional neural networks. Proceedings of the 2015 ACM/SIGDA International Symposium on Field-Programmable Gate Arrays.

[26] Neshatpour K., Mokrani H.M., Sasan A., Ghasemzadeh H., Rafatirad S., Homayoun H. Architectural considerations for FPGA acceleration of machine learning applications in MapReduce. Proceedings of the 18th International Conference on Embedded Computer Systems: Architectures, Modeling, and Simulation.

[27] Iandola F.N., Han S., Moskewicz M.W., Ashraf K., Dally W.J., Keutzer K. SqueezeNet: AlexNet-level Accuracy With 50× Fewer Parameters and <0.5 mb Model Size. http://arxiv.org/abs/1602.07360.

[28] Deng Y. Deep learning on mobile devices: A review. Proceedings of the SPIE 10993, Mobile Multimedia/Image Processing, Security, and Applications 2019, 109930A.

[29] Kim D., Ahn J., Yoo S. A novel zero weight/activation-aware hardware architecture of convolutional neural network. Proceedings of the 2017 Design, Automation and Test in Europe DATE 2017.

[30] Lecun Y., Bengio Y., Hinton G. (2015). Deep learning. Nature.

[31] Schmidhuber J. (2015). Deep learning in neural networks: An overview. Neural Netw..

[32] Jawandhiya P. (2018). Hardware design for machine learning. Int. J. Artif. Intell. Appl..

[33] Chen J., Ran X. (2019). Deep learning with edge computing: A review. Proc. IEEE.

[34] Frank M., Drikakis D., Charissis V. (2020). Machine-learning methods for computational science and engineering. Computation.

[35] Xiong Z., Zhang Y., Niyato D., Deng R., Wang P., Wang L.C. (2019). Deep reinforcement learning for mobile 5G and beyond: Fundamentals, applications, and challenges. IEEE Veh. Technol. Mag..

[36] Carbonell J.G. (1981). Machine learning research. ACM SIGART Bull..

[37] Jadhav S.D., Channe H.P. (2016). Comparative STUDY of K-NN, naive bayes and decision tree classification techniques. Int. J. Sci. Res..

[38] Chapter 4 Logistic Regression as a Classifier. https://www.cs.cmu.edu/~kdeng/thesis/logistic.pdf.

[39] Salvadori C., Petracca M., del Rincon J.M., Velastin S.A., Makris D. (2017). An optimisation of Gaussian mixture models for integer processing units. J. Real Time Image Process..

[40] Das A., Borisov N., Caesar M. Do you hear what i hear? Fingerprinting smart devices through embedded acoustic components. Proceedings of the ACM Conference on Computer, Communication and Security.

[41] Bojinov H., Michalevsky Y., Nakibly G., Boneh D. Mobile Device Identification via Sensor Fingerprinting. http://arxiv.org/abs/1408.1416.

[42] Huynh M., Nguyen P., Gruteser M., Vu T. Mobile device identification by leveraging built-in capacitive signature. Proceedings of the ACM Conference on Compututer, Communication and Security.

[43] Dhar S., Sreeraj K.P. (2015). FPGA implementation of feature extraction based on histopathalogical image and subsequent classification by support vector machine. IJISET Int. J. Innov. Sci. Eng. Technol..

[44] Yu L., Ukidave Y., Kaeli D. GPU-accelerated HMM for speech recognition. Proceedings of the International Conference Parallel Processing Work.

[45] Zubair M., Yoon C., Kim H., Kim J., Kim J. Smart wearable band for stress detection. Proceedings of the 2015 5th International Conference IT Converg. Secur. ICITCS.

[46] Razavi A., Valkama M., Lohan E.S. K-means fingerprint clustering for low-complexity floor estimation in indoor mobile localization. Proceedings of the 2015 IEEE Globecom Work. GC Wkshps 2015.

[47] Bhide V.H., Wagh S. I-learning IoT: An intelligent self learning system for home automation using IoT. Proceedings of the 2015 International Conference Communication Signalling Process. ICCSP 2015.

[48] Munisami T., Ramsurn M., Kishnah S., Pudaruth S. (2015). Plant Leaf recognition using shape features and colour histogram with K-nearest neighbour classifiers. Proc. Comput. Sci..

[49] Sowjanya K., Singhal A., Choudhary C. MobDBTest: A machine learning based system for predicting diabetes risk using mobile devices. Proceedings of the Souvenir 2015 IEEE Int. Adv. Comput. Conference IACC 2015.

[50] Lee J., Stanley M., Spanias A., Tepedelenlioglu C. Integrating machine learning in embedded sensor systems for Internet-of-Things applications. Proceedings of the 2016 IEEE International Symposium on Signal Processing and Information Technology (ISSPIT).

[51] Qiu J., Wang J., Yao S., Guo K., Li B., Zhou E., Yu J., Tang T., Xu N., Song S. Going deeper with embedded FPGA platform for convolutional neural network. Proceedings of the FPGA 2016ACM/SIGDA International Symposium Field-Programmable Gate Arrays.

[52] Huynh L.N., Balan R.K., Lee Y. DeepSense: A GPU-based deep convolutional neural network framework on commodity mobile devices. Proceedings of the Workshop on Wearable Systems and Application Co-Located with MobiSys 2016.

[53] Tuama A., Comby F., Chaumont M. Camera model identification based machine learning approach with high order statistics features. Proceedings of the 24th European Signal Processing Conference (EUSIPCO).

[54] Kurtz A., Gascon H., Becker T., Rieck K., Freiling F. (2016). Fingerprinting Mobile Devices Using Personalized Configurations. Proc. Priv. Enhanc. Technol..

[55] Mohsin M.A., Perera D.G. An FPGA-based hardware accelerator for k-nearest neighbor classification for machine learning on mobile devices. Proceedings of the ACM International Conference Proceeding Series, HEART 2018.

[56] Patil S.S., Thorat S.A. Early detection of grapes diseases using machine learning and IoT. Proceedings of the 2016 Second International Conference on Cognitive Computing and Information Processing (CCIP).

[57] Ollander S., Godin C., Campagne A., Charbonnier S. A comparison of wearable and stationary sensors for stress detection. Proceedings of the IEEE International Conference System Man, and Cybernetic SMC 2016.

[58] Moreira M.W.L., Rodrigues J.J.P.C., Oliveira A.M.B., Saleem K. Smart mobile system for pregnancy care using body sensors. Proceedings of the International Conference Sel. Top. Mob. Wirel. Networking, MoWNeT 2016.

[59] Shapsough S., Hesham A., Elkhorazaty Y., Zualkernan I.A., Aloul F. Emotion recognition using mobile phones. Proceedings of the 2016 IEEE 18th International Conference on e-Health Networking, Applications and Services (Healthcom).

[60] Hakim A., Huq M.S., Shanta S., Ibrahim B.S.K.K. (2016). Smartphone based data mining for fall detection: Analysis and design. Proc. Comput. Sci..

[61] Ronao C.A., Cho S.B. (2017). Recognizing human activities from smartphone sensors using hierarchical continuous hidden Markov models. Int. J. Distrib. Sens. Netw..

[62] Kodali S., Hansen P., Mulholland N., Whatmough P., Brooks D., Wei G.Y. Applications of deep neural networks for ultra low power IoT. Proceedings of the 35th IEEE International Conference on Computer Design ICCD 2017.

[63] Zhang X., Zhou X., Lin M., Sun J. ShuffleNet: An extremely efficient convolution neural network for mobile devices. Proceedings of the IEEE Conference on Computer Vision and Pattern Recognition (CVPR).

[64] Baldini G., Dimc F., Kamnik R., Steri G., Giuliani R., Gentile C. (2017). Identification of mobile phones using the built-in magnetometers stimulated by motion patterns. Sensors.

[65] Azimi I., Anzanpour A., Rahmani A.M., Pahikkala T., Levorato M., Liljeberg P., Dutt N. (2017). HiCH: Hierarchical fog-assisted computing architecture for healthcare IoT. ACM Trans. Embed. Comput. Syst..

[66] Pandey P.S. Machine Learning and IoT for prediction and detection of stress. Proceedings of the 17th International Conference on Computational Science and Its Applications ICCSA 2017.

[67] Sneha H.R., Rafi M., Kumar M.V.M., Thomas L., Annappa B. Smartphone based emotion recognition and classification. Proceedings of the 2nd IEEE International Conference on Electrical, Computer and Communication Technology ICECCT 2017.

[68] Al Mamun M.A., Puspo J.A., Das A.K. An intelligent smartphone based approach using IoT for ensuring safe driving. Proceedings of the 2017 International Conference on Electrical Engineering and Computer Science (ICECOS).

[69] Neyja M., Mumtaz S., Huq K.M.S., Busari S.A., Rodriguez J., Zhou Z. An IoT-based e-health monitoring system using ECG signal. Proceedings of the IEEE Global Communications Conference GLOBECOM 2017.

[70] Gupta C., Suggala A.S., Goyal A., Simhadri H.V., Paranjape B., Kumar A., Goyal S., Udupa R., Varma M., Jain P. ProtoNN: Compressed and accurate kNN for resource-scarce devices. Proceedings of the 34th International Conference on Machine Learning.

[71] Fafoutis X., Marchegiani L., Elsts A., Pope J., Piechocki R., Craddock I. Extending the battery lifetime of wearable sensors with embedded machine learning. Proceedings of the IEEE World Forum on Internet Things, WF-IoT 2018.

[72] Damljanovic A., Lanza-Gutierrez J.M. An embedded cascade SVM approach for face detection in the IoT edge layer. Proceedings of the IECON 2018—44th Annual Conference of the IEEE Industrial Electronics Society.

[73] Hochstetler J., Padidela R., Chen Q., Yang Q., Fu S. Embedded deep learning for vehicular edge computing. Proceedings of the 3rd ACM/IEEE Symposium on Edge Computing SEC 2018.

[74] Taylor B., Marco V.S., Wolff W., Elkhatib Y., Wang Z. (2018). Adaptive deep learning model selection on embedded systems. ACM SIGPLAN Not..

[75] Strielkina A., Kharchenko V., Uzun D. (2018). A markov model of healthcare internet of things system considering failures of components. CEUR Workshop Proc..

[76] Vhaduri S., van Kessel T., Ko B., Wood D., Wang S., Brunschwiler T. Nocturnal cough and snore detection in noisy environments using smartphone-microphones. Proceedings of the IEEE International Conference on Healthcare Informatics, ICHI 2019.

[77] Sattar H., Bajwa I.S., Amin R.U., Sarwar N., Jamil N., Malik M.A., Mahmood A., Shafi U. (2019). An IoT-based intelligent wound monitoring system. IEEE Access.

[78] Mengistu D., Frisk F. Edge machine learning for energy efficiency of resource constrained IoT devices. Proceedings of the Fifth International Conference on Smart Portable, Wearable, Implantable and Disabilityoriented Devices and Systems, SPWID 2019.

[79] Wang S., Tuor T., Salonidis T., Leung K.K., Makaya C., He T., Chan K. (2019). Adaptive Federated Learning in Resource Constrained Edge Computing Systems. IEEE J. Sel. Areas Commun..

[80] Suresh P., Fernandez S.G., Vidyasagar S., Kalyanasundaram V., Vijayakumar K., Archana V., Chatterjee S. (2020). Reduction of transients in switches using embedded machine learning. Int. J. Power Electron. Drive Syst..

[81] Giri D., Chiu K.L., di Guglielmo G., Mantovani P., Carloni L.P. ESP4ML: Platform-based design of systems-on-chip for embedded machine learning. Proceedings of the 2020 Design, Automation and Test in European Conference Exhibition DATE 2020.

[82] Tiku S., Pasricha S., Notaros B., Han Q. (2020). A hidden markov model based smartphone heterogeneity resilient portable indoor localization framework. J. Syst. Archit..

[83] Mazlan N., Ramli N.A., Awalin L., Ismail M., Kassim A., Menon A. (2020). A smart building energy management using internet of things (IoT) and machine learning. Test. Eng. Manag..

[84] Cornetta G., Touhafi A. (2021). Design and evaluation of a new machine learning framework for iot and embedded devices. Electronics.

[85] Rabiner L., Juang B. (1986). An introduction to hidden Markov models. IEEE ASSP Mag..

[86] Degirmenci A. (2014). Introduction to hidden markov models. Harv. Univ..

[87] Tóth B., Németh G. (2012). Optimizing HMM speech synthesis for low-resource devices. J. Adv. Comput. Intell. Intell. Inform..

[88] Fu R., Zhao Z., Tu Q. (2011). Reducing computational and memory cost for HMM-based embedded TTS system. Commun. Comput. Inf. Sci..

[89] Baoli L., Shiwen Y., Qin L. (2005). An improved K-nearest neighbor algorithm for text categorization. Dianzi Yu Xinxi Xuebao J. Electron. Inf. Technol..

[90] Norouzi M., Fleet D.J., Salakhutdinov R. (2012). Hamming distance metric learning. Adv. Neural Inf. Process. Syst..

[91] Saikia J., Yin S., Jiang Z., Seok M., Seo J.S. K-nearest neighbor hardware accelerator using in-memory computing SRAM. Proceedings of the 2019 IEEE/ACM International Symposium on Low Power Electronics and Design (ISLPED).

[92] Pedersen R., Schoeberl M. An embedded support vector machine. Proceedings of the 2006 International Workshop on Intelligent Solutions in Embedded Systems.

[93] You Y., Fu H., Song S.L., Randles A., Kerbyson D., Marquez A., Yang G., Hoisie A. (2015). Scaling support vector machines on modern HPC platforms. J. Parallel Distrib. Comput..

[94] Boni A., Pianegiani F., Petri D. (2007). Low-power and low-cost implementation of SVMs for smart sensors. IEEE Trans. Instrum. Meas..

[95] Afifi S.M., Gholamhosseini H., Sinha R. (2015). Hardware implementations of SVM on FPGA: A state-of-the-art review of current practice. Int. J. Innov. Sci. Eng. Technol..

[96] Zeng Z.Q., Yu H.B., Xu H.R., Xie Y.Q., Gao J. Fast training support vector machines using parallel sequential minimal optimization. Proceedings of the 2008 3rd International Conference on Intelligent System and Knowledge Engineering.

[97] Anguita D., Ghio A., Oneto L., Parra X., Reyes-Ortiz J.L. (2012). Human activity recognition on smartphones using a multiclass hardware-friendly support vector machine. Lect. Notes Comput. Sci..

[98] Kudo T., Matsumoto Y. Chunking with support vector machines. Proceedings of the Second Meeting of the North American Chapter of the Association for Computational Linguistics 2001.

[99] Osuna E., Freund R., Girosi F. Improved training algorithm for support vector machines. Neural Networks for Signal Processing VII. Proceedings of the 1997 IEEE Signal Processing Society Workshop.

[100] Lee Y.J., Mangasarian O. RSVM: Reduced Support vector machines. Proceedings of the Proceedings of the 2001 SIAM International Conference on Data Mining.

[101] Anguita D., Ghio A., Pischiutta S., Ridella S. A hardware-friendly support vector machine for embedded automotive applications. Proceedings of the 2007 International Joint Conference on Neural Networks.

[102] Anguita D., Bozza G. The effect of quantization on support vector machines with Gaussian kernel. Proceedings of the 2005 IEEE International Joint Conference on Neural Networks.

[103] Khan F.M., Arnold M.G., Pottenger W.M. Hardware-based support vector machine classification in logarithmic number systems. Proceedings of the 2005 IEEE International Symposium on Circuits and Systems.

[104] Anguita D., Pischiutta S., Ridella S., Sterpi D. (2006). Feed-forward support vector machine without multipliers. IEEE Trans. Neural Netw..

[105] Reynolds D. (2009). Gaussian mixture models. Encycl. Biometr..

[106] Gorur P., Amrutur B. Speeded up Gaussian mixture model algorithm for background subtraction. Proceedings of the 2011 8th IEEE International Conference on Advanced Video and Signal Based Surveillance (AVSS).

[107] Shen Y., Hu W., Liu J., Yang M., Wei B., Chou C.T. Efficient background subtraction for real-time tracking in embedded camera networks. Proceedings of the 10th ACM Conference on Embedded Networked Sensor System.

[108] Bottou L., Montavon G., Orr G.B., Müller K.R. (2012). Stochastic Gradient Descent Tricks. Neural Networks: Tricks of the Trade. Lecture Notes in Computer Science.

[109] Johnson R., Zhang T. (2013). Accelerating stochastic gradient descent using predictive variance reduction. Adv. Neural Inf. Process. Syst..

[110] Bottou L. (1991). Stochastic gradient learning in neural networks, Proc. Neuro-Nımes.

[111] Li L., Zhang S., Wu J. An efficient hardware architecture for activation function in deep learning processor. Proceedings of the 2018 IEEE 3rd International Conference on Image, Vision and Computing (ICIVC).

[112] Suda N., Chandra V., Dasika G., Mohanty A., Ma Y., Vrudhula S., Seo J.S., Cao Y. Throughput-optimized OpenCL-based FPGA Accelerator for large-scale convolutional neural networks. Proceedings of the ACM/SIGDA International Symposium on Field-Programmable Gate Arrays.

[113] Learning S.D. (2017). Smartphones devices. IEEE Pervasive Comput..

[114] Albawi S., Mohammed T.A., Al-Zawi S. Understanding of a convolutional neural network. Proceedings of the 2017 International Conference on Engineering and Technology (ICET).

[115] O’Shea K., Nash R. An Introduction to Convolutional Neural Networks. http://arxiv.org/abs/1511.08458.

[116] Lawrence S., Giles L., Tsoi C., Back A. (1997). Face recognition: A convolutional neural-network approach. IEEE Trans. Neural Netw..

[117] Hochreiter S., Schmidhuber J. (1997). Long Short-term memory. Neural Comput..

[118] Shah S., Haghi B., Kellis S., Bashford L., Kramer D., Lee B., Liu C., Andersen R., Emami A. Decoding kinematics from human parietal cortex using neural networks. Proceedings of the 2019 9th International IEEE/EMBS Conference on Neural Engineering (NER).

[119] Lee D., Lim M., Park H., Kang Y., Park J.S., Jang G.J., Kim J.H. (2017). Long short-term memory recurrent neural network-based acoustic model using connectionist temporal classification on a large-scale training corpus. Chin. Commun..

[120] Yu Y., Si X., Zhang J. (2019). A review of recurrent neural networks: LSTM cells and network architectures. Neural Comput..

[121] Khan M.A., Karim M.R., Kim Y. (2018). A two-stage big data analytics framework with real world applications using spark machine learning and long short-term memory network. Symmetry.

[122] Jouppi N.P., Young C., Patil N., Patterson D. (2018). A domain-specific architecture for deep neural networks. Commun. ACM.

[123] Zeiler M.D., Fergus R. (2014). Visualizing and understanding convolutional networks. Lect. Notes Comput. Sci..

[124] Han S., Pool J., Tran J., Dally W.J. (2015). Learning both weights and connections for efficient neural networks. Proceedings of the NIPS’15: Proceedings of the 28th International Conference on Neural Information Processing Systems.

[125] Khoram S., Li J. Adaptive quantization of neural networks. Proceedings of the 6th International Conference on Learning Representations (ICLR 2018).

[126] Al-Kofahi M.M., Al-Shorman M.Y., Al-Kofahi O.M. (2019). Toward energy efficient microcontrollers and Internet-of-Things systems. Comput. Electr. Eng..

[127] Keras A. Keras API Reference/Keras Applications. https://keras.io/api/applications/.

[128] Atmel ATMEL—ATmega48P/88P/168P/328P. https://www.sparkfun.com/datasheets/Components/SMD/ATMega328.pdf.

[129] Atmel Corporation ATMEL—ATmega640/V-1280/V-1281/V-2560/V-2561/V. https://ww1.microchip.com/downloads/en/devicedoc/atmel-2549-8-bit-avr-microcontroller-atmega640-1280-1281-2560-2561_datasheet.pdf.

[130] STMicroelectronics STM32L073x8 STM32L073xB. https://www.st.com/resource/en/datasheet/stm32l073v8.pdf.

[131] Atmel Corporation 32-Bit ARM-Based Microcontrollers SAM D21E/SAM D21G/SAM D21J Summary. www.microchip.com.

[132] Atmel SAM3X / SAM3A Series datasheet. http://www.atmel.com/Images/Atmel-11057-32-bit-Cortex-M3-Microcontroller-SAM3X-SAM3A_Datasheet.pdf.

[133] STMicroelectronics STM32F215xx STM32F217xx. https://www.st.com/resource/en/datasheet/stm32f215re.pdf.

[134] STMicroelectronics STM32F469xx. https://www.st.com/resource/en/datasheet/stm32f469ae.pdf.

[135] Raspberry Pi Dramble Power Consumption Benchmarks. https://www.pidramble.com/wiki/benchmarks/power-consumption.

[136] The First Affordable RISC-V Computer Designed to Run Linux. https://www.seeedstudio.com/blog/2021/01/13/meet-beaglev-the-first-affordable-risc-v-single-board-computer-designed-to-run-linux/.

[137] Lane N.D., Bhattacharya S., Georgiev P., Forlivesi C., Jiao L., Qendro L., Kawsar F. DeepX: A Software accelerator for low-power deep learning inference on mobile devices. Proceedings of the 2016 15th ACM/IEEE International Conference on Information Processing in Sensor Networks (IPSN).

[138] Li D., Wang X., Kong D. DeepRebirth: Accelerating deep neural network execution on mobile devices. Proceedings of the Thirty-Second AAAI Conference on Artificial Intelligence.

[139] Ren T.I., Cavalcanti G.D.C., Gabriel D., Pinheiro H.N.B., Yin H., Costa J.A.F., Barreto G. (2012). A Hybrid GMM Speaker Verification System for Mobile Devices in Variable Environments. Intelligent Data Engineering and Automated Learning—IDEAL 2012.

[140] Lei X., Senior A., Gruenstein A., Sorensen J. Accurate and compact large vocabulary speech recognition on mobile devices. Proceedings of the Annual Conference of the International Speech Communication Association INTERSPEECH.

[141] Sanchez-Iborra R., Skarmeta A.F. (2020). TinyML-enabled frugal smart objects: Challenges and opportunities. IEEE Circuits Syst. Mag..

[142] Park J., Naumov M., Basu P., Deng S., Kalaiah A., Khudia D., Law J., Malani P., Malevich A., Nadathur S. (2018). Deep learning inference in facebook data centers: Characterization, performance optimizations and hardware implications. arXiv.

[143] Banbury C., Zhou C., Fedorov I., Matas R., Thakker U., Gope D., Janapa Reddi V., Mattina M., Whatmough P. MicroNets: Neural network architectures for deploying TinyML Applications on commodity microcontrollers. Proceedings of the 4th MLSys Conference.

[144] NVIDIA NVIDIA V100 Tensor Core GPU. https://www.nvidia.com/en-us/data-center/v100/.

[145] NVIDIA The Ultimate PC GPU Nvidia Titan RTX. https://www.nvidia.com/content/dam/en-zz/Solutions/titan/documents/titan-rtx-for-creators-us-nvidia-1011126-r6-web.pdf.

[146] ST Microelectronics STM32F745xx STM32F746xx Datasheet. http://www.st.com/content/ccc/resource/technical/document/datasheet/96/ed/61/9b/e0/6c/45/0b/DM00166116.pdf/files/DM00166116.pdf/jcr:content/translations/en.DM00166116.pdf.

[147] ST Microelectronics Inc. STM32F765xx, STM32F767xx Datasheet. https://pdf1.alldatasheet.com/datasheet-pdf/view/933989/STMICROELECTRONICS/STM32F767ZI.html.

[148] Capra M., Bussolino B., Marchisio A., Shafique M., Masera G., Martina M. (2020). An Updated survey of efficient hardware architectures for accelerating deep convolutional neural networks. Future Internet.

[149] Sun S., Cao Z., Zhu H., Zhao J. (2020). A survey of optimization methods from a machine learning perspective. IEEE Trans. Cybern..

[150] Han S., Mao H., Dally W.J. Deep compression: Compressing deep neural networks with pruning, trained quantization and Huffman coding. Proceedings of the 4th International Conference on Learning Representations.

[151] Hubara I., Courbariaux M., Soudry D., El-Yaniv R., Bengio Y. (2018). Quantized neural networks: Training neural networks with low precision weights and activations. J. Mach. Learn. Res..

[152] Tanaka K., Arikawa Y., Ito T., Morita K., Nemoto N., Miura F., Terada K., Teramoto J., Sakamoto T. Communication-efficient distributed deep learning with GPU-FPGA heterogeneous computing. Proceedings of the 2020 IEEE Symposium on High-Performance Interconnects (HOTI).

[153] Lane N., Bhattacharya S., Georgiev P., Forlivesi C. (2017). Squeezing deep learning into mobile and embedded devices. IEEE Pervasive Comput..

[154] Gysel P. Ristretto: Hardware-Oriented Approximation of Convolutional Neural Networks. http://arxiv.org/abs/1605.06402.

[155] Moons B., Goetschalckx K., van Berckelaer N., Verhelst M. Minimum energy quantized neural networks. Proceedings of the 2017 51st Asilomar Conference on Signals, Systems, and Computers ACSSC 2017.

[156] Xu C., Kirk S.R., Jenkins S. Tiling for performance tuning on different models of GPUs. Proceedings of the 2009 Second International Symposium on Information Science and Engineering ISISE 2009.

[157] Sun F., Li X., Wang Q., Tang C. FPGA-based embedded system design. Proceedings of the IEEE Asia-Pacific Conference Circuits Systems APCCAS.

[158] Roth W., Schindler G., Zöhrer M., Pfeifenberger L., Peharz R., Tschiatschek S., Fröning H., Pernkopf F., Ghahramani Z. Resource-Efficient Neural Networks for Embedded Systems. http://arxiv.org/abs/2001.03048.

[159] Courbariaux M., Bengio Y., David J.P. Low Precision Storage for Deep Learning. http://arxiv.org/abs/1511.00363%5Cnhttp://arxiv.org/abs/1412.7024.

[160] Courbariaux M., David J.P., Bengio Y. Training deep neural networks with low precision multiplications. Proceedings of the 3rd International Conference on Learning Representations.

[161] Tong J.Y.F., Nagle D., Rutenbar R.A. (2000). Reducing power by optimizing the necessary precision/range of floating-point arithmetic. IEEE Trans. Very Large Scale Integr. Syst..

[162] Tagliavini G., Mach S., Rossi D., Marongiu A., Benin L. A transprecision floating-point platform for ultra-low power computing. Proceedings of the 2018 Design, Automation & Test in Europe Conference & Exhibition (DATE).

[163] Langroudi S.H.F., Pandit T., Kudithipudi D. Deep Learning inference on embedded devices: Fixed-point vs posit. Proceedings of the 2018 1st Workshop on Energy Efficient Machine Learning and Cognitive Computing for Embedded Applications (EMC2).

[164] Oberstar E. Fixed-Point Representation & Fractional Math. http://www.superkits.net/whitepapers/Fixed%20Point%20Representation%20&%20Fractional%20Math.pdf.

[165] Yates R. Fixed-point arithmetic: An introduction. Technical Reference. https://courses.cs.washington.edu/courses/cse467/08au/labs/l5/fp.pdf.

[166] Hwang K., Sung W. Fixed-point feedforward deep neural network design using weights +1, 0, and −1. Proceedings of the 2014 IEEE Workshop on Signal Processing Systems (SiPS).

[167] Gupta S., Agrawal A., Gopalakrishnan K., Narayanan P. Deep learning with limited numerical precision. Proceedings of the 32nd International Conference on Machine Learning ICML 2015.

[168] Gustafson J.L., Yonemoto I. (2017). Beating floating point at its own game: Posit arithmetic. Supercomput. Front. Innov..

[169] Hammerstrom D. A VLSI architecture for high-performance, low-cost, on-chip learning. Proceedings of the IJCNN. International JT Conference Neural Network.

[170] Courbariaux M., Hubara I., Soudry D., El-Yaniv R., Bengio Y. Binarized Neural Networks: Training Deep Neural Networks with Weights and Activations Constrained to +1 or −1. http://arxiv.org/abs/1602.02830.

[171] Meng W., Gu Z., Zhang M., Wu Z. Two-Bit Networks for Deep Learning on Resource-Constrained Embedded Devices. http://arxiv.org/abs/1701.00485.

[172] Park E., Ahn J., Yoo S. Weighted-entropy-based quantization for deep neural networks. Proceedings of the IEEE Conference on Computer Vision and Pattern Recognition (CVPR).

[173] Burrascano P. (1991). Learning vector quantization for the probabilistic neural network. IEEE Trans. Neural Netw..

[174] Mittal A., Tiku S., Pasricha S. Adapting convolutional neural networks for indoor localization with smart mobile devices. Proceedings of the 2018 on Great Lakes Symposium on VLSI, 2018; GLSVLSI’18.

[175] Hu R., Tian B., Yin S., Wei S. Efficient hardware architecture of softmax layer in deep neural network. Proceedings of the 2018 IEEE 23rd International Conference on Digital Signal Processing (DSP).

[176] Hennessy J.L., Patterson D.A. (2019). A new golden age for computer architecture. Commun. ACM.

[177] Kim R.G., Doppa J.R., Pande P.P., Marculescu D., Marculescu R. (2018). Machine learning and manycore systems design: A Serendipitous symbiosis. Computer.

[178] Kim R.G., Doppa J.R., Pande P.P. Machine learning for design space exploration and optimization of manycore systems. Proceedings of the 2018 IEEE/ACM International Conference on Computer-Aided Design (ICCAD).

[179] Vazquez R., Gordon-Ross A., Stitt G. Machine learning-based prediction for dynamic architectural optimizations. Proceedings of the 10th International Green and Sustainability Computing Conference IGSC 2019.

[180] Papp D., Ma Z., Buttyan L. Embedded systems security: Threats, vulnerabilities, and attack taxonomy. Proceedings of the 2015 13th Annual Conference on Privacy, Security and Trust (PST).

[181] Ogbebor J.O., Imoize A.L., Atayero A.A.-A. (2020). Energy Efficient Design Techniques in Next-Generation Wireless Communication Networks: Emerging Trends and Future Directions. Wirel. Commun. Mob. Comput..

[182] Imoize A.L., Ibhaze A.E., Atayero A.A., Kavitha K.V.N. (2021). Standard Propagation Channel Models for MIMO Communication Systems. Wirel. Commun. Mob. Comput..

[183] Popoola S.I., Jefia A., Atayero A.A., Kingsley O., Faruk N., Oseni O.F., Abolade R.O. (2019). Determination of neural network parameters for path loss prediction in very high frequency wireless channel. IEEE Access.

[184] Faruk N., Popoola S.I., Surajudeen-Bakinde N.T., Oloyede A.A., Abdulkarim A., Olawoyin L.A., Ali M., Calafate C.T., Atayero A.A. (2019). Path loss predictions in the VHF and UHF bands within urban environments: Experimental investigation of empirical, heuristics and geospatial models. IEEE Access.

[185] Pasricha S., Nikdast M. (2020). A Survey of Silicon Photonics for Energy-Efficient Manycore Computing. IEEE Des. Test.

[186] Soref R. (2006). The past, present, and future of silicon photonics. IEEE J. Sel. Top. Quantum Electron..

[187] Chittamuru S.V.R., Dang D., Pasricha S., Mahapatra R. (2018). BiGNoC: Accelerating big data computing with application-specific photonic network-on-chip architectures. IEEE Trans. Parallel Distrib. Syst..

